# High Fat Diet-Induced Changes in Hepatic Protein Abundance in Mice

**DOI:** 10.4172/jpb.1000214

**Published:** 2012-02-29

**Authors:** Moulun Luo, April E. Mengos, Tianna M. Stubblefield, Lawrence J. Mandarino

**Affiliations:** 1 Center for Metabolic and Vascular Biology, Mayo Clinic Arizona, Scottsdale, Arizona; Arizona State University, Tempe, Arizona, USA; 2 Department of Medicine, Mayo Clinic Arizona, Scottsdale, Arizona, USA

**Keywords:** C57BL/6J, High fat diet, Insulin resistance, NAFLD, Mass spectrometry

## Abstract

Nonalcoholic fatty liver disease (NAFLD) is associated with obesity, insulin resistance, type 2 diabetes, and dyslipidemia. The purpose of this study was to identify novel proteins and pathways that contribute to the pathogenesis and complications of NAFLD. C57BL/6J male mice were fed a 60% (HFD) or 10% (LFD) high or low fat diet. HFD induced obesity, hepatic steatosis and insulin resistance (euglycemic clamps, glucose infusion rate: LFD 50.5 ± 6.4 vs. HFD 14.2 ± 9.5 μg/ (g·min); *n* = 12). Liver proteins were analyzed by mass spectrometry-based proteomics analysis. Numerous hepatic proteins were altered in abundance after 60% HFD feeding. Nine down-regulated and nine up-regulated proteins were selected from this list for detailed analysis based on the criteria of 1.5-fold difference, consistency across replicates, and having at least 2 spectra assigned. Proteins that decreased in abundance were acyl-coA desaturase-I (SCD-1), acetyl-CoA carboxylase (ACC), fatty acid synthase (FAS), pyruvate kinase isozymes R/L (PKLR), NADP-dependent malic enzyme (ME-1), ATP-citrate synthase (ACL), ketohexokinase (KHK), long-chain-fatty acid-CoA ligase-5 (ACSL-5) and carbamoyl-phosphate synthase-I (CPS-1). Those that increased were KIAA0564, apolipoprotein A-I (apoA-1), ornithine aminotransferase (OAT), multidrug resistance protein 2 (MRP-2), liver carboxylesterase-I (CES-1), aminopeptidase N (APN), fatty aldehyde dehydrogenase (FALDH), major urinary protein 2 (MUP-2) and KIAA0664. KIAA0564 and KIAA0664 proteins are uncharacterized and are novel proteins associated with NAFLD. The decreased abundance of normally highly abundant proteins like FAS and CPS-1 was confirmed by Coomassie Blue staining after bands were identified by MS/MS, and immunoblot analysis confirmed the increased abundance of KIAA0664 after 60% HFD feeding. In conclusion, this study shows NAFLD is characterized by changes in abundance of proteins related to cell injury, inflammation, and lipid metabolism. Two novel and uncharacterized proteins, KIAA0564 and KIAA0664, may provide insight into the pathogenesis of NAFLD induced by lipid oversupply.

## Introduction

Type 2 diabetes mellitus is the most common metabolic disease and is one of the leading causes of death in the United States. Insulin resistance plays an important role in the development of the disease. Of several organs that exhibit insulin resistance, liver is one of the most important. Liver has a wide range of metabolic functions, including gluconeogenesis, glycogenesis, cholesterol biosythesis, lipogenesis, protein synthesis and detoxification. Excessive ectopic lipid accumulation in the liver, or non-alcoholic fatty liver disease (NAFLD), is closely associated with obesity, insulin resistance and type 2 diabetes [1–3]. Stefan et al. [4] noted that, in a cohort of obese people, insulin-sensitive and insulin-resistant individuals could be distinguished on the basis of lipid accumulation in muscle and liver, rather than subcutaneous or visceral adiposity. Fabbrini et al. [5] also reported that intrahepatic triglyceride content, not visceral adiposity was associated with insulin resistance. Although some hypotheses, including the involvement of inflammatory mechanisms [6,7] and the DAG-PKC pathway [8] have been raised to explain the mechanism of hepatic insulin resistance, they remain to be fully explored.

The most common animal model used for investigating the mechanism by which insulin resistance develops is the feeding of male C57BL/6J mice with a high-fat diet (HFD) resulting in the development of obesity, NAFLD and insulin resistance [8]. Moreover, long-term high fat feeding coupled with a methionine and choline-free diet can induce nonalcoholic steatohepatitis (NASH) in C57BL/6J mice [9]. Gene expression changes in insulin resistant liver have been investigated by microarray analysis and Real Time RT-PCR, using the high fat diet-induced model of insulin resistance in mice, Several genes, such as fatty acid synthase (FAS), Acetyl-CoA carboxylase (ACC) and stearoyl CoA desaturase (SCD) were highlighted [10,11]. Other genes involved in metabolism, transcription, signaling and defense/inflammation mechanism have also been studied by analyzing changes in mRNA expression [10–12].

Although mRNA expression is a useful parameter for the evaluation of gene transcription changes in liver, extensive analysis of protein expression and relative abundance has not been forthcoming. It can be argued that changes in protein abundance may be a closer reflection of changes in liver function than mRNA levels. In this study, we used mass spectrometry to identify proteins in liver tissues and compared the resultant number of spectra between mice fed a low fat diet (LFD) and a high fat diet (HFD). We found that the abundance of numerous hepatic proteins was altered in the livers of high fat diet fed mice. We hypothesize that some of these proteins might be involved in cell injury, inflammation mechanisms, the DAG-PKC pathway or other pathways, resulting in NAFLD and insulin resistance.

## Materials and Methods

### Animals and diets

All procedures were approved by the Mayo Clinic Institutional Animal Care and Use Committee (Protocol No. A38109). Male, 6 week old C57BL/6J mice were purchased from Harlan Teklad (Houston, TX) and housed under controlled temperature (23°C) and lighting (10 h light:14 h dark) conditions with free access to water and food. Animals were placed on an irradiated rodent diet (10% fat diet D12450B; or 60% fat diet D12492, Research Diets, Brunswick, NJ) for 9 weeks before surgery. Body composition was assessed by magnetic resonance spectroscopy using Bruker Minispec LF90II (Bolerica, MA). One week before clamp procedures, surgical implantation of a silastic catheter into the right jugular vein was performed in preparation for clamp infusions. Surgical anesthesia was maintained by sodium pentobarbital (i.p. 60 mg/kg body wt). The free catheter ends were tunneled under the skin to the back of the neck and attached via stainless steel connectors to tubing made of Micro-Renathane; the tubing was externalized and sealed with stainless steel plugs. Animals were individually housed after surgery. Animals not within 10% of their presurgery weight by post surgery day 5 were excluded from the study.

### Hyperinsulinemic-euglycemic clamps

After being fasted for 5 hours, mice were placed in a restrainer (552-BSRR; Plas-Labs, Lansing, MI) for the duration of the clamp procedure. Blood was collected at set time points via cut tail sampling. The clamp protocol consisted of a 90-min tracer equilibration period (*t* = –90 to 0 min) beginning at 11:30 AM followed by a 120-min clamp experimental period (*t* = 0 to 120 min) beginning at 1:00 PM. Mice received saline-washed erythrocytes from donors throughout the clamp period (3.5 μl/min) to prevent a fall of >5% hematocrit. A blood sample (about 5 μl) was obtained at *t* = −90 min to determine initial glucose levels (One Touch UltraSmart, LifeScan, Milpitas, CA). A 5-μCi bolus of [3–3H] glucose was given at *t* = −90 min followed by a 0.05 μCi/ min infusion for 90 minutes. At *t* = −5 min, a blood sample (100 μl) was taken for the assessment of basal glucose and insulin levels and glucose turnover. The insulin clamp began at *t* = 0 min with a continuous infusion of human insulin (2.5 mU kg–1 min–1; Humulin R; Eli Lilly, Indianapolis, IN). The [3-^3^H] glucose infusion was increased to 0.1 μCi/ min for the remainder of the experiment in order to minimize changes in specific activity from the equilibration period. Thus, the slope of specific activity over time would not vary significantly from zero. Euglycemia (150–160 mg/dl) was maintained during the clamp by measuring blood glucose every 10 min starting at *t* = 0 min and infusing 50% dextrose as necessary. Blood samples (60–100 μl) were taken every 10 min from *t* = 90 to 120 min and processed to determine glucose specific activity. Clamp insulin levels were determined from samples obtained at *t* = 100 and 120 min. At the end of the clamps, mice were anesthetized with pentobarbital sodium (jugular vein infusion, 60 mg/kg) and tissues were harvested, flash frozen, and stored in liquid nitrogen for subsequent analysis.

### Metabolic studies

Mice were feed with 10% LFD or 60% HFD diet for 10 weeks and placed into Oxymax/CLAMS (Columbus Instruments, Columbus, HO) with free access to food and water, allowing them to acclimate in individual metabolic cages for 16 h before any measurements. Subsequently, 72-h metabolic profiles were generated in successive 26-min cycles. VO_2_ was expressed as milliliters per kilogram per minute. Studies were performed at 22°C. The sensor was calibrated against a standard gas mix containing defined quantities of O_2_ and CO_2_. Oxygen consumption (VO_2_), CO_2_ production, respiratory exchange ratio (RER), food intake and spontaneous locomotor activity were continuously measured using the system The cumulative amount of food eaten during 72 hours was recorded. Animal activity was calculated via the animal interruption of a photocell beam in either X, Y or Z direction that then accrues to one “count”.

### Histology

Paraffin-embedded sections of liver were stained by hematoxylin and eosin and periodic acid Schiff/diastase to examine cellular architecture and lipid accumulation.

### Mass spectrometry

For sample preparation, mouse liver tissues were homogenized and the homogenates were resolved by SDS-PAGE. The gel bands were excised and digested with trypsin. The digested peptides were subjected to HPLC-ESI-MS/MS analysis. For mass spectrometry, data analysis and bioinformatics, all were performed as described previously [13,14]. Only peptides with ≥ 95% probability based on Scaffold analysis were considered. Criteria for protein identification include detection of at least 2 unique peptides and a probability score of ≥ 99%.

### Label-free quantification of protein abundance

In order to compare the abundance of a protein between different samples, the numbers of MS/MS spectra assigned to the protein in each sample needs to be normalized by NSAF (normalized spectral abundance factor), as was described previously [15]. The NSAF calculation is simplified by replacing the number of amino acids with the molecular weight. For a protein *i*, the normalized spectral abundance factor, NSAF, is calculated by

$$NSAFi=SAFi/\sum_{i=1}^{N}SAFi\;\text{Where}\;\text{SAF}i=(\text{spectrum}\;\text{count})i÷(\text{molecular}\;\text{weight})i$$

In this study, since more than 1000 proteins were identified in mouse liver by mass spectrometry, only the proteins which responded with the largest changes in abundance were selected for further investigation. The selection criteria used to include a protein for detailed analysis in this study was first, that the ratio of the number of spectra for a given protein between treatment and control was at least 1.5 or more, or 0.7 or less, second, that the trend of change (increase or decrease) for the protein of interest remained consistent across triplicate pairs (control vs. corresponding treatment), and third, that either sample in each pair comparison had at least two spectra for the protein of interest.

### Antibodies and immunoblot analysis

KIAA0664 antibody was purchased from US Biological (Swampscott, MA). For immunoblotting, proteins were separated by SDS-PAGE, transferred onto nitrocellulose membranes, and detected with primary antibody followed by horseradish peroxidase-conjugated secondary antibodies.

## Results

### High fat diet feeding results in obesity and insulin resistance in mice

In order to identify hepatic proteins that respond to the consumption of a high dietary fat, and which may potentially be responsible for the development of insulin resistance, six week old male C57BL/6J mice were fed a 10% low fat diet (LFD, control) or a 60% high fat diet (HFD) for 9 weeks. At the end of this period, body composition was assessed using NMR and insulin sensitivity was quantified using euglycemic, hyperinsulinemic clamps on conscious mice. At the end of each glucose clamp, mouse tissues were harvested and stored in liquid nitrogen for further analysis. Mice fed with a 60% high fat diet, compared to the control 10% fat diet group, have increased body weight, body fat, liver weight (although liver weight to body weight ratio did not differ) and a decreased rate of glucose infusion required to maintain euglycemia (Table 1), confirming that the high fat diet feeding caused obesity and insulin resistance. In order to rule out any potentially spurious results arising from stress due to surgeries and clamp procedures, two additional control groups of six week old male C57BL/6J mice were fed a 10% low fat diet (LFD, control) or a 60% high fat diet (HFD) for 10 weeks, but were not subjected to surgeries or clamps before being weighed, analyzed for body fat percentage, and sacrificed for tissue collection. These two “non-clamped” groups, as compared to the two groups which underwent surgery and clamps, were of slightly higher body weight and had larger livers, but did not differ in percentage of body fat. The HFD resulted in a lower respiratory exchange ratio (RER) and less spontaneous activity (Table 2). Although total food intake (grams) was lower in the HFD mice (Table 2), the caloric content of the food eaten was not significantly different (23.8 ± 2.9 vs. 19.6 ± 2.7 kCal, LFD vs. HFD). Regardless, the HFD mice still consumed almost 6 times more fat calories.

### High fat diet feeding results in obesity and hepatic steatosis in mice

**Figure 1:**
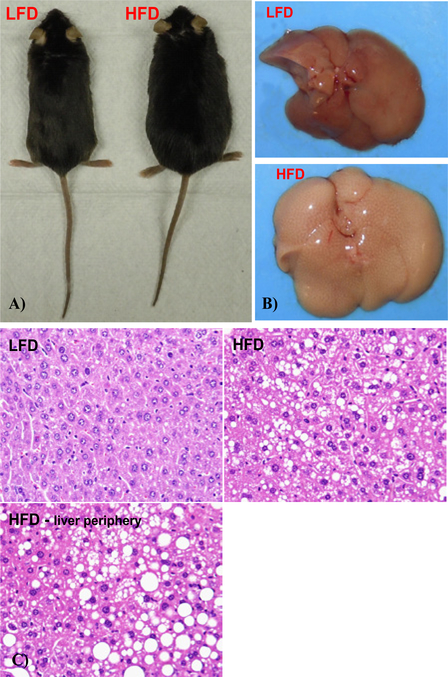
High fat diet consumption leads to obesity and hepatic steatosis. A. Representative low fat diet group (LFD) mouse and high fat diet group (HFD) mouse. B. Representative LFD mouse liver and HFD mouse liver. C. Representative liver sections stained with hematoxylin and eosin (original magnification 400×) showing the increased size of lipid droplets in HFD liver over that of the LFD group, especially on the periphery of the organ.

After the 10 week feeding regimen, the mice in the 60% HFD group had greater body weight than those in the 10% LFD group (Figure 1A). The livers of the 60% HFD mice were pale in color as compared to the healthy red color observed in 10% LFD mice (Figure 1B). Extensive hepatic steatosis developed in the livers of 60% HFD mice, with more advanced accumulation of lipid droplets on the periphery of the organ (Figure 1C). The data clearly indicate that mice in the 60% HFD group have developed obesity and hepatic steatosis.

### Identification of hepatic proteins that respond to the consumption of elevated dietary fat

**Figure 2:**
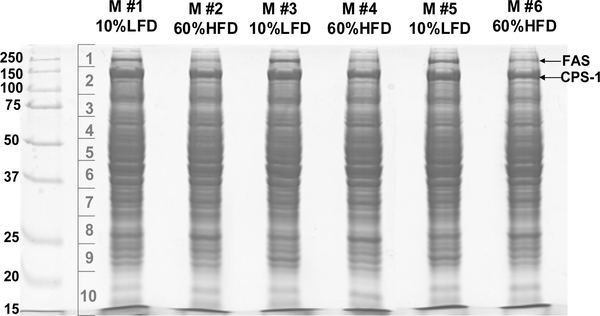
SDS-PAGE for identification of hepatic proteins via mass spectrometry analysis. The mouse liver tissues were homogenized and the proteins were resolved by SDS-PAGE. The protein bands were excised at 10 slices per gel lane and digested by trypsin *in situ.* The digested peptides were subjected to HPLC-ESI-MS/MS analysis.

To identify hepatic proteins that change in abundance in response to high fat feeding, mouse livers harvested after the glucose clamp were homogenized and the proteins were resolved by SDS-PAGE (Figure 2). The protein bands were excised at 10 slices per gel lane and digested by trypsin *in situ.* The digested peptides were subjected to HPLC-ESI-MS/MS analysis. Supplemental Table 1 shows the numbers of MS/MS spectra assigned to each protein, as well as NSAF values for each protein. Using the selection criteria described under Label-free quantification of protein abundance of Material and Methods, nine down-regulated proteins and nine up-regulated proteins were selected for detailed analysis. Table 3 shows selected proteins that were down-regulated by a high fat diet, including acyl-coA desaturase-I (SCD-1), acetyl-CoA carboxylase (ACC), fatty acid synthase (FAS), pyruvate kinase isozymes R/L (PKLR), NADP-dependent malic enzyme (ME-1), ATP-citrate synthase (ACL), ketohexokinase (KHK), long-chain-fatty acid-CoA ligase-5(ACSL-5) and carbamoyl-phosphate synthase-I (CPS-1). Table 4 shows the selected up-regulated proteins, including apolipoprotein A-I (Apo A-1), ornithine aminotransferase (OAT), multidrug resistance protein 2 (MRP-2), liver carboxylesterase-I (CES-1), amino peptidase N (APN), fatty aldehyde dehydrogenase (FALDH), major urinary protein 2 (MUP-2), KIAA0564, and KIAA0664.

**Figure 3:**
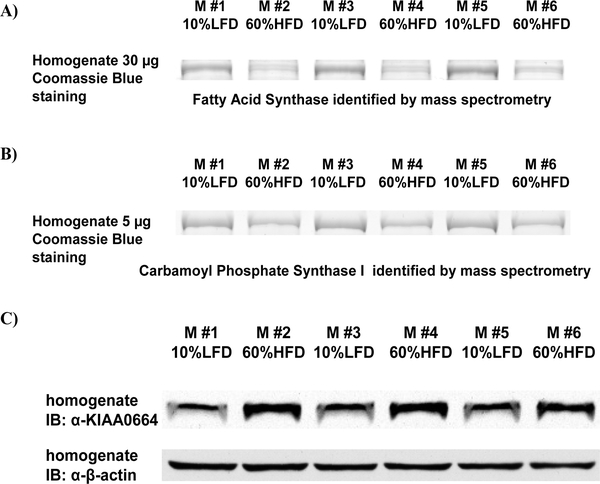
Decreased expression of FAS and CPS-1, and increased expression of KIAA0664 in mouse liver due to high fat feeding were confirmed by corroborative methods. The mouse liver tissues were homogenized and the proteins were resolved by SDS-PAGE. A. For FAS, the gel was stained with Coomassie Blue and the bands were validated as FAS by mass spectrometry. B. For CPS-1, the gel was stained with Coomassie Blue and the bands were validated as CPS-1 by mass spectrometry. C. For KIAA0664, the gel was subjected to Western blotting and probed with anti-KIAA0664 and anti-beta actin antibodies.

To confirm that the expression level of Fatty Acid Synthase (FAS) and Carbamoyl-phosphate synthase I (CPS-1) are decreased in mouse liver upon high fat feeding, mouse liver tissues were homogenized and the proteins were resolved by SDS-PAGE. The gels were stained with Coomassie Blue and the bands shown in Figure 3A and 3B were positively identified as FAS and CPS-1 by mass spectrometry. The intensity of the Coomassie Blue stained bands demonstrate that FAS and CPS-1 have decreased expression in the livers of 60%HFD mice as compared to that of 10%LFD mice. To confirm that the expression level of the uncharacterized protein KIAA0664 is increased in mouse liver due to high fat feeding, the mouse liver homogenates were subjected to immunoblotting. Figure 3C shows that KIAA0664 protein is more abundantly expressed in the livers of 60%HFD mice than in those of 10%LFD mice.

In addition to the nine up-regulated and nine down-regulated proteins above, three proteins Sec24a (13.4 fold increase of NSAF in 60%HFD), glutathione S-transferase (4.4 fold increase in HFD), and derlin-2 (3.2 fold increase in HFD), which are involved in ER oxidative stress, all were increased upon high fat feeding (see Supplemental Table 1).

## Discussion

Nonalcoholic fatty liver disease (NAFLD) is a continuum of disease that includes simple steatosis (nonalcoholic fatty liver) and nonalcoholic steatohepatitis (NASH) that develops in the absence of excessive alcohol intake and includes fibrosis. NAFLD and NASH ultimately can lead to the development of cirrhosis and hepatocellular carcinoma (HCG) [16]. Multiple studies have defined the following clinical features to be associated with the development of NAFLD and NASH; overweight and obesity, insulin resistance, type 2 diabetes, hypertension and dyslipidemia [17].

As a means of studying NAFLD, animal models have been developed that imitate the Western-style, high fat diet. Mice fed a diet comprised of 60% fat (60% HFD) developed obesity, hepatic steatosis and insulin resistance, an increase reliance on fat as an oxidative substrate (lower RER), and lower spontaneous physical activity. To investigate the mechanism by which obesity, hepatic steatosis and insulin resistance are induced by a high fat diet, approaches involving gene and protein expression analysis have been attempted to track changes on the molecular level in liver in response to elevated dietary fat consumption [18,19]. In this study, we identified a number of hepatic proteins that are changed in abundance in mice with high fat diet induced obesity, hepatic steatosis and insulin resistance. Nine down-regulated proteins and nine up-regulated proteins responsive to high fat feeding were selected for more detailed analysis. The selection criteria used were detailed in Materials and Methods. Because the liver pathology in the high fat fed mouse resembles fatty liver (NAFLD) rather than NASH, it should be cautioned that the protein changes here should be viewed in that context.

The current results show that in response to a high fat diet, a number of hepatic proteins that are involved in lipogenesis were down-regulated, possibly due to feedback mechanisms. Acetyl-CoA carboxylase (ACC), fatty acid synthase (FAS) and stearyl CoA desaturase (SCD) are three primary enzymes involved in lipogenesis in liver. Table 3 shows that ACC, FAS and Acyl-coA desaturase-1 (SCD-1, a member of stearyl CoA desaturase) all are lower in abundance in the livers of 60% HFD mice. The decreases in abundance of ACC and FAS found in this study are consistent with published reports where mRNA levels of ACC and FAS, and FAS protein abundance were decreased after high fat diet feeding [10,20,21]. However, the reports, where SCD-1 mRNA was shown to be increased upon high fat diet feeding [11,18], are inconsistent with our results and illustrate the importance of assessing protein abundance changes. Another lipogenic enzyme, ATP-citrate synthase (aka ATP-citrate lyase, ACL), was decreased in abundance in liver due to high fat feeding, which is also consistent with published data [21]. Other enzymes involved in lipogenesis, namely NADP-dependent malic enzyme (ME-1) and long-chain-fatty acid-CoA ligase-5 (ACSL-5), also had decreased expression in livers of 60% HFD mice. Furthermore, it has been reported that ME-1 mRNA levels decrease in response to a high fat diet, which is consistent with our results [10, 20]. Taken together, the data support the assertion that a feedback mechanism activated due to lipid oversupply is suppressing the expression of enzymes involved in lipogenesis. However, down-regulation of these proteins was insufficient to completely compensate for the elevated fat content in the diet, as evidence by the presence of excess ectopic fat in livers of HFD mice.

It could be speculated that at the same time proteins involved in lipogenesis are down-regulated, proteins involved in lipolysis and lipid oxidation might be up-regulated in livers of HFD mice. However, our data show that only liver carboxylesterase I, which is involved in lipolysis, was increased in abundance (Table 4). Therefore, although the liver appears well adapted to decrease lipogenesis in the face of lipid oversupply, it may be less able to increase its utilization of lipid. This imbalance in the adaptability of liver to lipid supply may contribute to ectopic lipid accumulation and contribute to liver disease. Another protein involved in lipid metabolism, apolipoprotein A-1 (apoA-1) was increase after HFD in this study. ApoA-1 is a component of high density lipoprotein particles. It has been reported that apoA-1 abundance increases in the livers of high-fructose-fed hamsters [22], similar to our result in HFD mice, suggesting this protein is responsive to excess caloric consumption. It also may imply that, to the extent liver contributes to whole body RER, any increase in fat oxidation was accounted for by the higher supply of lipid.

In addition to lipogenic enzymes, glycolytic enzymes were down-regulated in liver in response to a high fat diet. Decreases in pyruvate kinase isozymes R/L and ketohexokinase abundance indicate glycolysis is also suppressed in the livers of HFD mice (Table 3). This also appears to reflect a compensatory response, as the need for glucose as fuel, or as lipogenic substrate would be reduced under HFD conditions.

Major urinary proteins (MUPs) are members of the lipocalins family. They are secreted into circulation by the liver and excreted in urine. MUPs form a characteristic glove shape, encompassing a cup-like pocket that binds pheromones - molecular signals excreted by one individual that trigger an innate behavioral response in another member of the same species. Recently, MUP-1 has identified as a regulator for glucose and lipid metabolism in mice [23,24], although the mechanism is unclear. It could be speculated that changes in MUP expression in response to diet changes could serve as molecular signals to other animals.

The current results show that high fat feeding suppressed the expression of hepatic proteins involved in protein and nitrogen metabolism. The changes observed potential could contribute to cell injury and inflammation due to the accumulation of toxic levels of ammonia. Mass spectrometry analysis revealed that Carbamoyl-phosphate synthase I (CPS-1) may be the most abundant protein in mouse liver (see Figure 2 and Table 3; the prominent CPS-1 band was associated with the largest number of assigned spectra and highest NSAF). Although the CPS-1 HFD/LFD ratio fell outside of the selection criteria for a decreased protein of interest (ratio greater than 0.7) CPS-1 was still taken into consideration because it was the most abundant protein found in liver in our study, yielding adequate number of spectra to lend statistical significance to the comparison between its expression in HFD and LFD fed mice. CPS-1 plays a vital role in protein and nitrogen metabolism. Optimal function of the urea cycle is dependent on adequate CPS-1 expression, and deficiency of CPS-1 causes abnormal nitrogen accumulation in tissues in the form of ammonia [25]. Excess ammonia is highly toxic to the body. In a recent publication, CPS-1 expression has been reported to be suppressed in the livers of high-fructose-fed hamsters [22]. Similarly, CPS-1 abundance is decreased in the livers of our 60% HFD mice. If this scenario were correct, such a change in protein abundance could contribute to liver damage in NAFLD.

Increases in abundance of three other proteins contribute to a potential mechanism for liver damage induced by the consumption of excess dietary fat Ornithine aminotransferase (OAT) is a potential target for the treatment of hyperammonemias [26]. Amino peptidase N (APN, also known as CD13) has multiple functions and has consequently been designated to be a “moonlighting ectoenzyme [27]. APN is involved in cholesterol turnover and has now become a target for cancer chemotherapy [28]. Finally, fatty aldehyde dehydrogenase (FALDH) is related to detoxification processes [29].

Results of this study show that at least two uncharacterized proteins are altered by high fat feeding. KIAA0564 and KIAA0664 abundance were up-regulated due to high fat feeding. KIAA0564 is composed of 1905 amino acids and contains a predicted ATP binding site, AAA+ and AAA domains at its N-terminus, and a vWFA (Von Willebrand factor type A) domain at its C-terminus. The AAA and AAA+ proteins are involved in a range of processes, including DNA replication, protein degradation, membrane fusion, microtubule severing, peroxisome biogenesis, signal transduction and the regulation of gene expression. The vWFA domain also is found in proteins that are involved in a wide range of important cellular functions, including basal membrane formation, cell migration, cell differentiation, adhesion, haemostasis, signaling, chromosomal stability, malignant transformation and immune defenses. KIAA0664 exists as both a long (1353 amino acids) and short (1315 amino acids) isoform. These isoforms are identical except that the long version has an extra 38 amino acids at its N-terminus. BLAST analysis reveals that KIAA0664 has some homology with eIF-3 (eukaryotic translation initiation factor 3) of other species. However, alignment of mouse KIAA0664 with mouse eIF-3 (from A to S) reveals that there is poor identity between the two. The functions of KIAA0564 and KIAA0664 and the mechanisms by which these two uncharacterized proteins are up-regulated remains obscure and needs further investigation. It also must be cautioned that changes in protein abundance were assessed in livers of mice that had been fed the high or low fat diets and that also underwent a euglycemic clamp (insulin infusion), that may have had some effects on protein abundance. However, 2 hours is little time to observe the magnitude of changes in protein abundance we observed; moreover, both groups of mice had an identical insulin infusion. We also performed immunoblots for KIAA0664 on liver lysates from high fat and low fat fed mice that did not have an insulin infusion, and found an identical increase in this protein.

It has been reported that obesity also induces ER stress, and this, in turn, plays a central role in the development of insulin resistance and diabetes by triggering JNK activity [30]. In this study, increased abundance of Sec24a, glutathione S-transferase, and derlin-2 may suggest that ER stress contribute to the insulin resistance in the high fat fed mice.

In summary, our results show that a 60% high fat diet (HFD) induces obesity, hepatic steatosis and insulin resistance in male C57BL/6J mice. Mass spectrometry analysis identified a number of hepatic proteins that are changed in abundance in mice after 60% HFD feeding. Nine down-regulated proteins and nine up-regulated proteins that respond to the consumption of elevated dietary fat were selected for detailed analysis. Two of these proteins, KIAA0564 and KIAA0664, are at present uncharacterized; hence, they are of special interest for future study. In conclusion, the identity of hepatic proteins that are changed in abundance in mice upon high fat feeding may potentially provide new information about the processes involved in the development of NAFLD and insulin resistance. We hypothesize that these mechanisms are related to cell injury, inflammation, and various other pathways.

## Supplementary Material

Supplemental Table 1

## Figures and Tables

**Table 1:** High fat diet results in obesity and insulin resistance in mice.

Clamped mice	LFD	HFD
*n*	12	12
Body Weight (g)	29.0 ±1.6	40.3 ± 3.8**
Body Fat (%)	14.2 ± 3.8	31.9 ± 2.3**
Liver Weight (g)	1.13 ± 0.13	1.45 ± 0.40*
Liver Weight/ Body Weight (g Liver/30g Body)	1.16 ± 0.08	1.06 ± 0.22
Glucose Infusion Rate (mg/min.kg)	50.5 ± 6.4	14.2 ± 9.5**

* p<0.05

** p<0.01 vs. LFD

**Table 2:** High fat diet results in obesity in mice (non-clamped).

Non-clamped mice	LFD	HFD
*n*	7	7
Body Weight (g)	33.3 ± 2.1	46.7 ± 2.9**
Body Fat (%)	18.7 ± 1.7	30.2 ± 1.1**
Respiratory Exchange Ratio	0.87 ± 0.02	0.72 ± 0.05**
Food Intake (g)	6.18 ± 0.76	3.73 ± 0.52**
Animal Activity (counts)	816 ± 80	488 ± 35**
Liver Weight (g)	1.59 ± 0.22	2.47 ± 0.50**
Liver Weight/ Body Weight (g Liver/ 30g Body)	1.43 ± 0.12	1.57 ± 0.23

* p<0.05

** p<0.01vs. LFD

**Table 3:** Identification of hepatic proteins responsive to a high fat diet (selected down-regulated).

		Spectra assigned to the protein						NSAF			
Down-regulated proteins	M W	M#1 10% LFD	M#2 60% HFD	M#3 10% LFD	M#4 60% HFD	M#5 10% LFD	M#6 60% HFD	M±SE 10% LFD	M±SE 60% HFD	$\frac{\text{HFD}}{\text{LFD}}$ Ratio	Function
Acyl-CoA desaturase 1	41kD	3	0	3	0	9	1	6.08±4.42	0.43±0.74	0.07	Lipogenesis
Acetyl-CoA carboxylase 1	265kD	23	8	38	4	31	9	5.68±1.34	1.36±0.53*	0.24	Lipogenesis
Fatty acid synthase	272kD	158	51	214	43	215	76	35.39±6.32	10.70±3.56*	0.30	Lipogenesis
Pyruvate kinase isozymes R/L	62kD	32	9	35	11	34	11	26.68±1.45	8.52±1.07*	0.32	Glycolysis
NADP-dependent malic enzyme	64kD	16	8	11	5	25	7	13.43±5.95	5.33±1.24	0.40	Lipogenesis
ATP-citrate synthase	120kD	23	10	33	12	29	11	11.60±2.01	4.68±0.43*	0.40	Lipogenesis
Ketohexokinase	33kD	24	8	17	7	22	12	31.33±5.85	14.01±4.53*	0.45	Glycolysis
Long-chain-fatty-acid- CoA ligase 5	76kD	14	2	12	9	13	7	8.41±0.76	4.04±2.42	0.48	Lipogenesis
Carbamoyl-phosphate synthase 1	165kD	573	426	562	441	464	395	158.4±13.3	130.1±4.1*	0.82	Urea cycle

Data for NSAF are value x10,000.

* p<0.05,vs.10%LFD.

**Table 4:** Identification of hepatic proteins responsive to a high fat diet (selected up-regulated).

		Spectra assigned to the protein						NSAF			
Up-regulated proteins	M W	M#1 10% LFD	M#2 60% HFD	M#3 10% LFD	M#4 60% HFD	M#5 10% LFD	M#6 60% HFD	M±SE 10% LFD	M±SE 60% HFD	$\frac{\text{HFD}}{\text{LFD}}$ Ratio	Function
Uncharacterised protein KIAA 0564	213kD	13	24	13	20	16	29	3.24±0.51	5.86±1.24*	1.81	Unknown
Apo lipoprotein A-1	31kD	9	12	2	8	6	10	9.03±5.57	16.49±3.29*	1.83	HDL Component
Ornithine aminotransferase	48kD	9	16	8	16	9	14	8.88±0.82	16.30±0.81*	1.84	Liver injury biomarker
Major urinary protein 2	21kD	17	46	32	70	46	67	74.6±36.2	148.6±33.2*	1.99	Lipid transport
Liver carboxylesterase 1	63kD	3	12	2	3	2	5	1.82±0.43	5.39±3.75	2.96	Lipolysis
Aminopeptidase N	110kD	4	9	3	6	1	8	1.18±0.66	3.56±1.73*	3.02	Kidney injury biomarker
Fatty aldehyde dehydrogenase	54kD	2	6	2	6	2	9	1.82±0.06	6.98±2.41*	3.83	Detoxification
Multidrug resistance protein 2	140kD	1	6	2	3	1	8	0.46±0.19	2.08±0.97	4.48	Bile salt transport
Uncharacterised protein KIAA 0664	148kD	2	14	5	10	0	12	0.76±0.81	4.14±0.69*	5.47	Unknown

Data for NSAF are value x10,000.

* p<0.05,vs.10%LFD

## Abbreviations

LFD: Low Fat Diet
HFD: High Fat Diet
NAFLD: Nonalcoholic Fatty Liver Disease
NASH: Nonalcoholic Steatohepatitis
HPLC-ESI-MS/MS: High Performance Liquid Chromatography/Electrospray Ionization/Mass Spectrometry
