# Supplementary material for: High Fat Diet-Induced Changes in Hepatic Protein Abundance in Mice
Source: J Proteomics Bioinform. Author manuscript; Available in PMC 2021 Apr 26. (PMC8074682; doi:10.4172/jpb.1000214)
Supplement: Supplemental Table 1 [file NIHMS955135-supplement-Supplemental_Table_1.pdf]

Supplemental Table 1. Relative abundance of hepatic proteins in 10%FAT and 60%FAT diet mice

Identified Proteins (1302)	M W (kD)	Spectra assigned to the protein M#1 10% FAT	M#2 60% FAT	M#3 10% FAT	M#4 60% FAT	M#5 10% FAT	M#6 60% FAT	NSAF Mean 10% FAT	SEM 10% FAT	Mean 60% FAT	SEM 60% FAT	Ratio 60%/10%
Cytochrome c oxidase subunit 5B, mitochondrial	14	7	0	3	0	0	0	11.52	12.21	0.00	0.00	0.00
Retinol dehydrogenase 11	35	0	0	3	0	3	0	2.82	2.45	0.00	0.00	0.00
Mitochondrial import inner membrane translocase subunit Tim17-B	18	0	0	1	0	2	0	2.77	2.83	0.00	0.00	0.00
60S ribosomal protein L19	23	1	0	1	0	3	0	3.61	2.63	0.00	0.00	0.00
UPF0556 protein C19orf10 homolog	18	4	0	2	0	0	0	5.37	5.41	0.00	0.00	0.00
Polyribonucleotide nucleotidyltransferase 1, mitochondrial	86	0	0	2	0	4	0	1.16	1.19	0.00	0.00	0.00
NFX1-type zinc finger-containing protein 1	219	0	0	0	0	3	0	0.23	0.40	0.00	0.00	0.00
Acetyl-coenzyme A synthetase, cytoplasmic	79	2	0	1	0	2	0	1.04	0.38	0.00	0.00	0.00
Eukaryotic translation initiation factor 1	13	2	0	1	0	0	0	3.72	3.74	0.00	0.00	0.00
Aminopeptidase B	72	1	0	1	0	3	0	1.15	0.84	0.00	0.00	0.00
ATP synthase subunit delta, mitochondrial	18	2	0	3	0	0	0	4.46	4.07	0.00	0.00	0.00
Acyl-CoA-binding protein	10	3	0	2	0	0	0	8.05	7.41	0.00	0.00	0.00
Proteasome subunit beta type-10	29	1	0	0	0	2	0	1.73	1.76	0.00	0.00	0.00
Macrophage migration inhibitory factor	13	3	0	1	0	0	0	4.97	5.72	0.00	0.00	0.00
Bile salt sulfotransferase 1	33	0	0	2	0	0	0	0.97	1.67	0.00	0.00	0.00
UV excision repair protein RAD23 homolog B	44	2	0	1	0	0	0	1.10	1.11	0.00	0.00	0.00
Metaxin-1	36	1	0	0	0	2	0	1.39	1.42	0.00	0.00	0.00
Prostaglandin E synthase 2	43	1	0	0	0	3	0	1.56	1.82	0.00	0.00	0.00
Cytochrome c oxidase subunit 6A1, mitochondrial	12	3	0	0	0	0	0	4.05	7.02	0.00	0.00	0.00
GTP cyclohydrolase 1 feedback regulatory protein	10	4	0	0	0	0	0	6.49	11.23	0.00	0.00	0.00
Adrenodoxin, mitochondrial	20	1	0	2	0	0	0	2.40	2.39	0.00	0.00	0.00
Cytochrome c oxidase subunit 6C	8	2	0	1	0	0	0	6.05	6.08	0.00	0.00	0.00
Leukotriene-B4 omega-hydroxylase 3	60	1	0	2	0	0	0	0.80	0.80	0.00	0.00	0.00
Copper homeostasis protein cutC homolog	29	2	0	1	0	0	0	1.67	1.68	0.00	0.00	0.00
Dephospho-CoA kinase domain-containing protein	26	0	0	1	0	2	0	1.92	1.96	0.00	0.00	0.00
ER lumen protein retaining receptor 1	25	0	0	1	0	2	0	2.00	2.04	0.00	0.00	0.00

Golgi resident proteinGCP60	60	0	0	1	0	2	0	0.83	0.85	0.00	0.00	0.00
NADH dehydrogenase [ubiquinone] 1 alpha subcomplex subunit 13	17	2	0	1	0	0	0	2.85	2.86	0.00	0.00	0.00
26S proteasome non-ATPase regulatory subunit 4	41	1	0	0	0	2	0	1.22	1.24	0.00	0.00	0.00
Serine-threonine kinase receptor-associated protein	38	1	0	0	0	2	0	1.32	1.34	0.00	0.00	0.00
5-formyltetrahydrofolate cyclo-ligase	23	2	0	0	0	0	0	1.41	2.44	0.00	0.00	0.00
60S ribosomal protein L35	15	2	0	0	0	0	0	2.16	3.74	0.00	0.00	0.00
Gamma-glutamylcyclotransferase	21	0	0	0	0	3	0	2.43	4.21	0.00	0.00	0.00
ATP synthase subunit f, mitochondrial	10	2	0	0	0	0	0	3.24	5.62	0.00	0.00	0.00
10 kDa heat shock protein, mitochondrial	11	2	0	0	0	0	0	2.95	5.11	0.00	0.00	0.00
Casein kinase II subunit beta	25	2	0	0	0	0	0	1.30	2.25	0.00	0.00	0.00
Dolichol-phosphate mannosyltransferase	29	2	0	0	0	0	0	1.12	1.94	0.00	0.00	0.00
Heat shock 70 kDa protein 1A	70	0	0	0	0	2	0	0.49	0.84	0.00	0.00	0.00
Guanylate kinase	22	2	0	0	0	0	0	1.47	2.55	0.00	0.00	0.00
Probable carboxypeptidase PM20D1	56	2	0	0	0	0	0	0.58	1.00	0.00	0.00	0.00
39S ribosomal protein L19, mitochondrial	34	0	0	0	0	2	0	1.00	1.73	0.00	0.00	0.00
39S ribosomal protein L21, mitochondrial	23	0	0	0	0	2	0	1.48	2.56	0.00	0.00	0.00
Small nuclear ribonucleoprotein Sm D3	14	2	0	0	0	0	0	2.32	4.01	0.00	0.00	0.00
Acyl-CoA desaturase 1	41	3	0	3	0	9	1	6.08	4.42	0.43	0.74	0.07
60S ribosomal protein L23	15	4	0	4	1	0	0	8.57	7.43	1.12	1.93	0.13
Asparaginyl-tRNA synthetase, cytoplasmic	64	4	1	2	0	1	0	1.78	1.15	0.26	0.45	0.15
D-dopachrome decarboxylase	13	9	1	5	1	0	0	17.36	16.86	2.58	2.23	0.15
ATP synthase subunit g, mitochondrial	11	9	2	5	0	0	0	20.51	19.93	3.05	5.29	0.15
E3 UFM1-protein ligase 1	90	3	0	1	0	3	1	1.28	0.65	0.20	0.34	0.15
Tumor protein D54	24	2	0	4	0	1	1	4.72	2.98	0.73	1.27	0.16
26S proteasome non-ATPase regulatory subunit 8	30	2	1	2	0	2	0	3.28	0.11	0.56	0.97	0.17
60S ribosomal protein L15	24	4	2	5	0	3	0	8.15	1.79	1.40	2.42	0.17
T-complex protein 1 subunit zeta	58	0	1	4	0	2	0	1.69	1.65	0.29	0.50	0.17
DnaJ homolog subfamily A member 1	45	3	0	2	0	1	1	2.17	1.06	0.39	0.68	0.18
Ras-related protein Rab-2A	24	4	0	3	2	4	0	7.53	1.36	1.40	2.42	0.19
Phosphotriesterase-related protein	39	4	1	4	0	3	1	4.61	0.59	0.88	0.76	0.19

40S ribosomal protein S11	18	2	0	1	1	2	0	4.58	1.67	0.93	1.61	0.20
Cytochrome P450 2J5	58	2	0	2	1	1	0	1.40	0.45	0.29	0.50	0.21
Transcription elongation factor B polypeptide 2	13	2	1	2	0	1	0	6.25	2.02	1.29	2.24	0.21
Glycerol kinase	61	1	0	3	1	1	0	1.33	0.89	0.27	0.48	0.21
40S ribosomal protein S19	16	3	1	2	0	0	0	5.03	4.63	1.05	1.82	0.21
Methylglutaconyl-CoA hydratase, mitochondrial	33	2	0	2	0	1	1	2.46	0.80	0.53	0.92	0.22
Protein LYRIC	64	2	0	2	0	1	1	1.27	0.41	0.27	0.48	0.22
Peroxiredoxin-4	31	2	0	3	1	4	1	4.78	1.73	1.11	0.96	0.23
Acetyl-CoA carboxylase 1	265	23	8	38	4	31	9	5.68	1.34	1.36	0.53	0.24
Nucleotide-binding protein-like	34	1	0	2	1	1	0	1.91	0.78	0.49	0.85	0.26
Eukaryotic initiation factor 4A-II	46	1	0	2	1	1	0	1.41	0.58	0.36	0.63	0.26
Cysteine and histidine-rich domain-containing protein 1	37	2	1	2	0	0	0	1.74	1.51	0.45	0.79	0.26
Ras GTPase-activating protein-binding protein 2	54	1	0	0	0	3	1	1.24	1.45	0.33	0.56	0.26
40S ribosomal protein S15a	15	2	1	6	1	0	0	8.54	9.73	2.24	1.94	0.26
Fatty acid-binding protein, liver	14	58	17	48	10	4	1	126.7	98.89	33.60	28.76	0.27
Nicotinate phosphoribosyltransferase	58	0	0	2	0	2	1	1.14	0.99	0.30	0.52	0.27
Prefoldin subunit 3	22	2	0	1	0	1	1	2.97	1.26	0.80	1.38	0.27
FAD-linked sulfhydryl oxidase ALR	23	2	0	1	0	1	1	2.84	1.20	0.76	1.32	0.27
Elongation factor G, mitochondrial	84	2	0	1	0	1	1	0.78	0.33	0.21	0.36	0.27
Profilin-1	15	4	2	3	0	0	0	7.51	6.72	2.24	3.88	0.30
26S proteasome non-ATPase regulatory subunit 3	61	1	0	3	1	3	1	1.89	0.95	0.56	0.49	0.30
60S ribosomal protein L31	14	3	2	4	0	0	0	8.03	7.14	2.40	4.15	0.30
26S proteasome non-ATPase regulatory subunit 11	47	2	0	1	0	4	2	2.48	1.70	0.75	1.29	0.30
Fatty acid synthase	272	158	51	214	43	215	76	35.39	6.32	10.70	3.56	0.30
Glucosidase 2 subunit beta	59	5	1	3	2	2	0	2.76	1.23	0.85	0.85	0.31
Glycerol-3-phosphate acyltransferase 1, mitochondrial	94	3	1	5	2	5	1	2.27	0.63	0.72	0.30	0.32
Pyruvate kinase isozymes R/L	62	32	9	35	11	34	11	26.69	1.45	8.52	1.07	0.32
40S ribosomal protein S13	17	6	3	6	0	1	1	12.35	8.10	4.00	4.51	0.32
Cytochrome P450 3A11	58	0	0	0	1	3	0	0.88	1.52	0.29	0.50	0.33
Actin-related protein 2/3 complex subunit 1B	41	1	1	0	0	2	0	1.22	1.24	0.41	0.71	0.33
Translation initiation factor eIF-2B subunit alpha	34	0	0	1	1	2	0	1.47	1.50	0.49	0.85	0.34
Eukaryotic translation initiation factor 4E	25	0	0	1	1	2	0	2.00	2.04	0.67	1.16	0.34
Dynamin-2	98	0	1	1	0	2	0	0.51	0.52	0.17	0.30	0.34

Carbohydrate kinase domain-containing protein	37	2	0	1	2	3	0	2.69	1.42	0.91	1.57	0.34
Histone H1.0	21	1	2	2	0	3	0	4.72	2.49	1.60	2.77	0.34
Twinfilin-1	40	2	0	0	1	1	0	1.24	1.22	0.42	0.73	0.34
Protein SCO1 homolog, mitochondrial	32	2	1	0	0	1	0	1.54	1.52	0.52	0.91	0.34
Putative helicase MOV-10	114	0	1	2	0	1	0	0.43	0.42	0.15	0.26	0.34
Translocon-associated protein subunit gamma	21	0	0	0	0	3	1	2.43	4.21	0.84	1.45	0.34
Splicing factor U2AF 65 kDa subunit	54	3	1	0	0	0	0	0.90	1.56	0.31	0.54	0.35
Peroxisomal membrane protein 11A	28	2	2	3	0	1	0	3.47	1.65	1.20	2.08	0.35
Isobutyryl-CoA dehydrogenase, mitochondrial	45	2	0	1	1	3	1	2.21	1.17	0.76	0.66	0.35
40S ribosomal protein S24	15	2	0	1	1	0	0	3.22	3.24	1.12	1.93	0.35
40S ribosomal protein S20	13	2	0	1	1	0	0	3.72	3.74	1.29	2.23	0.35
Histone H4	11	4	1	2	1	0	0	8.79	8.85	3.05	2.64	0.35
Glutathione reductase, mitochondrial	54	2	1	1	0	0	0	0.90	0.90	0.31	0.54	0.35
Cytochrome c, somatic	12	5	2	4	1	0	0	12.07	10.67	4.19	4.20	0.35
Bcl-2-like protein 13	47	1	0	2	1	0	0	1.02	1.02	0.36	0.62	0.35
Thioredoxin	12	1	1	2	0	0	0	4.01	3.98	1.40	2.42	0.35
Farnesyl pyrophosphate synthase	41	1	0	0	0	2	1	1.22	1.24	0.43	0.74	0.35
Microtubule-associated protein RP/EB family member 1	30	1	0	0	0	2	1	1.67	1.70	0.59	1.01	0.35
Phosphatidylinositol transfer protein beta isoform	31	0	0	1	0	2	1	1.61	1.65	0.57	0.98	0.35
Plasma protease C1 inhibitor	56	2	0	0	0	1	1	0.88	0.87	0.31	0.54	0.36
Alanine aminotransferase 2	58	1	0	2	0	0	1	0.83	0.82	0.30	0.52	0.37
Ribonuclease UK114	14	20	8	11	3	0	0	35.69	34.78	13.18	14.54	0.37
Glucose-6-phosphate isomerase	63	6	4	15	4	12	4	8.58	3.52	3.24	0.09	0.38
Chloride intracellular channel protein 4	29	5	2	4	1	2	1	6.17	2.46	2.34	0.98	0.38
Acylamino-acid-releasing enzyme	82	3	1	2	2	3	0	1.60	0.38	0.61	0.61	0.38
Rab GDP dissociation inhibitor alpha	51	2	2	3	1	3	0	2.57	0.58	0.99	0.99	0.38
Ras-related protein Rab-8A	24	1	2	4	0	3	1	5.46	3.08	2.13	2.10	0.39
Rho GDP-dissociation inhibitor 1	23	7	2	6	2	3	2	11.31	4.20	4.44	0.12	0.39
Ras-related protein Rab-1B	22	1	1	4	0	3	2	5.95	3.35	2.36	2.40	0.40
NADP-dependent malic enzyme	64	16	8	11	5	25	7	13.43	5.95	5.33	1.24	0.40
40S ribosomal protein S18	18	4	1	4	1	0	1	7.15	6.19	2.84	0.08	0.40
T-complex protein 1 subunit epsilon	60	4	3	5	0	4	2	3.54	0.39	1.42	1.29	0.40

Signal recognition particle 68 kDa protein	71	1	1	1	1	3	0	1.17	0.85	0.47	0.41	0.40
ATP-citrate synthase	120	23	10	33	12	29	11	11.60	2.01	4.68	0.43	0.40
Acidic leucine-rich nuclear phosphoprotein 32 family member A	29	3	2	2	2	5	0	5.71	2.81	2.31	2.00	0.41
E3 ubiquitin-protein ligase HUWE1	483	1	0	0	1	4	1	0.17	0.22	0.07	0.06	0.41
3-hydroxyisobutyryl-CoA hydrolase, mitochondrial	43	5	3	4	2	6	1	5.74	1.34	2.36	1.14	0.41
Ubiquinone biosynthesis protein COQ7 homolog	24	1	0	1	1	3	1	3.46	2.52	1.43	1.24	0.41
1-acyl-sn-glycerol-3-phosphate acyltransferase beta	31	1	2	3	0	1	0	2.61	1.74	1.08	1.88	0.41
40S ribosomal protein S16	16	3	1	2	1	0	0	5.03	4.63	2.10	1.82	0.42
Protein CDV3	30	2	0	1	1	2	1	2.75	1.00	1.14	0.99	0.42
40S ribosomal protein S26	13	2	0	3	2	0	0	6.17	5.63	2.58	4.46	0.42
Acyl-coenzyme A thioesterase 13	15	2	1	3	1	0	0	5.35	4.88	2.24	1.94	0.42
Inositol monophosphatase 1	30	1	0	2	1	2	1	2.74	0.97	1.14	0.99	0.42
Alpha-1-antitrypsin 1-2	46	3	1	1	0	1	1	1.77	1.21	0.75	0.65	0.42
ATP-binding cassette sub-family E member 1	67	1	1	3	0	1	1	1.21	0.81	0.51	0.44	0.42
Thioredoxin-like protein 1	32	4	1	7	1	4	4	7.64	2.45	3.24	2.90	0.42
Vesicle-associated membrane protein-associated protein B	27	2	1	3	0	0	1	2.97	2.71	1.27	1.10	0.43
Cytochrome c oxidase subunit 4 isoform 1, mitochondrial	20	4	3	8	2	0	0	9.62	9.56	4.19	3.84	0.44
Hexaprenyldihydroxybenzoate methyltransferase, mitochondrial	41	2	2	2	1	3	0	2.81	0.80	1.23	1.23	0.44
GrpE protein homolog 1, mitochondrial	24	4	2	4	0	4	3	8.19	0.27	3.59	3.33	0.44
Bisphosphoglycerate mutase	30	2	0	1	2	4	1	3.88	2.66	1.70	1.68	0.44
Putative deoxyribose-phosphate aldolase	35	1	1	2	1	4	1	3.32	2.28	1.46	0.04	0.44
Estradiol 17-beta-dehydrogenase 2	42	3	1	2	1	2	1	2.73	0.65	1.22	0.03	0.45
Thioesterase superfamily member 4	26	4	1	3	2	0	0	4.33	3.88	1.93	1.93	0.45
Ketohexokinase	33	24	8	17	7	22	12	31.33	5.85	14.01	4.53	0.45
Alpha-1-antitrypsin 1-5	46	2	2	4	0	1	1	2.46	1.55	1.11	1.10	0.45
Interferon-inducible GTPase1	48	4	4	2	0	3	0	3.08	1.04	1.40	2.42	0.45
Mesencephalic astrocyte-derived neurotrophic factor	20	4	4	6	1	1	0	8.87	5.94	4.19	5.24	0.47
Aldehyde dehydrogenase family 16 member A1	85	3	1	5	2	3	2	2.11	0.61	1.00	0.36	0.48
Elongation factor 1-beta	25	5	2	6	5	4	0	9.79	1.66	4.69	5.06	0.48

Long-chain-fatty-acid--CoA ligase 5	76	14	2	12	9	13	7	8.41	0.76	4.04	2.42	0.48
60S ribosomal protein L26	17	5	0	2	4	8	3	14.64	9.19	7.04	6.23	0.48
Stress-induced-phosphoprotein 1	63	3	3	6	1	4	2	3.37	1.12	1.62	0.80	0.48
Cytochrome P450 2D9	57	6	3	5	3	4	1	4.30	0.78	2.07	0.99	0.48
UPF0568 protein C14orf166 homolog	28	7	2	6	3	2	2	8.68	4.47	4.25	0.99	0.49
Zinc finger ZZ-type and EF-hand domain-containing protein 1	328	0	0	0	1	2	0	0.10	0.18	0.05	0.09	0.49
Transmembrane protein 126A	22	0	0	0	1	2	0	1.55	2.68	0.76	1.32	0.49
39S ribosomal protein L18, mitochondrial	21	0	1	0	0	2	0	1.62	2.80	0.80	1.38	0.49
Nuclear pore membrane glycoprotein 210	204	1	1	1	1	2	0	0.32	0.15	0.16	0.14	0.51
rRNA 2'-O-methyltransferase fibrillarin	34	1	2	1	0	2	0	1.95	0.91	0.99	1.71	0.51
Thioredoxin reductase 1, cytoplasmic	67	1	1	2	2	3	0	1.48	0.78	0.75	0.75	0.51
Splicing factor, arginine/serine-rich 7	31	1	1	2	2	3	0	3.20	1.69	1.62	1.62	0.51
Proteasome subunit alpha type-3	28	1	2	2	1	3	0	3.54	1.87	1.80	1.80	0.51
Coatomer subunit epsilon	35	4	2	1	2	3	0	3.77	2.16	1.92	1.66	0.51
GDP-L-fucose synthase	36	2	1	1	1	1	0	1.82	0.77	0.93	0.81	0.51
Splicing factor 3B subunit 3	136	0	1	1	0	3	1	0.49	0.58	0.25	0.22	0.51
26S proteasome non-ATPase regulatory subunit 13	43	2	2	1	0	1	0	1.52	0.64	0.78	1.35	0.51
Protein disulfide-isomerase A6	48	9	5	3	2	4	1	5.45	3.24	2.81	2.16	0.52
Lysyl-tRNA synthetase	68	3	2	2	1	1	0	1.43	0.70	0.74	0.74	0.52
Programmed cell death protein 6	22	2	0	0	1	2	1	3.02	2.62	1.56	1.35	0.52
Signal recognition particle receptor subunit alpha	70	0	0	0	0	2	1	0.49	0.84	0.25	0.43	0.52
Nucleolar protein 16	21	0	0	0	0	2	1	1.62	2.80	0.84	1.45	0.52
Mannose-binding protein C	26	2	1	0	0	2	1	2.55	2.21	1.32	1.14	0.52
60S ribosomal protein L27	16	3	2	1	0	0	0	4.04	4.65	2.10	3.63	0.52
Threonine synthase-like 2	54	2	3	4	0	2	1	2.41	0.98	1.26	1.42	0.52
Cytochrome c oxidase subunit 5A, mitochondrial	16	2	2	2	0	0	0	4.02	3.48	2.10	3.63	0.52
ATP-binding cassette sub-family A member 8-B	183	4	0	1	2	1	1	0.53	0.46	0.28	0.27	0.52
Peptidyl-prolyl cis-trans isomerase FKBP3	25	2	1	1	0	3	2	3.97	2.10	2.08	2.11	0.52
Alpha-soluble NSF attachment protein	33	2	2	3	0	3	2	3.98	0.90	2.08	1.80	0.52
Lipopolysaccharide-responsive and beige-like anchor protein	317	1	0	2	1	3	2	0.31	0.16	0.16	0.17	0.52

Histone H3.1	15	1	1	3	1	0	0	4.27	4.86	2.24	1.94	0.52
Eukaryotic translation initiation factor 3 subunit L	67	2	1	1	0	1	1	0.98	0.41	0.51	0.44	0.53
Transmembrane emp24 domain-containing protein 5	26	0	0	2	1	0	0	1.23	2.12	0.64	1.12	0.53
NADH dehydrogenase [ubiquinone] 1 beta subcomplex subunit 5, mitochondrial	22	0	1	2	0	0	0	1.45	2.51	0.76	1.32	0.53
6-phosphogluconate dehydrogenase, decarboxylating	53	3	1	1	0	2	2	1.86	0.93	0.98	0.99	0.53
Ras-related protein Rab-5A	24	1	0	2	1	1	1	2.71	1.10	1.43	1.24	0.53
Ras-related protein Rab-21	24	1	0	2	1	1	1	2.71	1.10	1.43	1.24	0.53
Atlastin-3	61	1	2	5	0	0	1	1.57	2.07	0.84	0.83	0.53
Fibrinogen beta chain	55	6	7	12	4	7	2	7.41	2.68	3.99	2.27	0.54
Immunity-related GTPase family M protein	47	1	0	2	0	1	2	1.38	0.56	0.75	1.29	0.54
Pyridoxal-dependent decarboxylase domain-containing protein 1	87	2	0	0	0	0	1	0.37	0.65	0.20	0.35	0.54
Hemoglobin subunit beta-1	16	78	40	58	27	6	7	143.2	112.1	77.91	51.76	0.54
Haptoglobin	39	6	4	6	4	5	1	7.13	0.51	3.89	2.20	0.55
Haloacid dehalogenase-like hydrolase domain-containing protein 3	28	2	3	4	2	5	1	6.47	2.83	3.62	1.76	0.56
Glycogen [starch] synthase, liver	81	4	1	4	2	5	4	2.64	0.44	1.49	1.01	0.56
Isochorismatase domain-containing protein 1	32	0	3	5	2	4	0	4.61	4.03	2.62	2.40	0.57
Epidermal growth factor receptor	135	13	9	9	2	11	7	4.01	0.76	2.28	1.36	0.57
Microsomal glutathione S-transferase 1	18	13	9	21	8	1	2	31.25	26.69	17.79	10.43	0.57
Fructose-bisphosphate aldolase A	39	1	0	4	4	4	1	3.79	2.21	2.17	2.67	0.57
Bifunctional ATP-dependent dihydroxyacetone kinase/ FAD-AMP lyase (cyclizing)	60	40	18	36	23	38	22	31.14	2.12	17.90	2.42	0.57
Endonuclease G, mitochondrial	32	3	1	2	2	4	2	4.64	1.69	2.67	0.95	0.58
Membrane-associated progesterone receptor component 2	23	3	1	4	3	2	1	6.36	1.94	3.68	2.49	0.58
Glycerol-3-phosphate dehydrogenase [NAD+], cytoplasmic	38	16	9	16	8	18	11	21.59	2.23	12.59	2.41	0.58
Transmembrane protein 111	30	3	1	1	2	3	1	3.85	1.96	2.26	0.94	0.59
Sorting nexin-2	58	4	3	1	0	2	1	1.98	1.28	1.17	1.32	0.59
UPF0585 protein C16orf13 homolog	23	7	2	2	3	5	3	10.02	5.40	5.94	1.36	0.59

60S acidic ribosomal protein P1	11	4	2	3	2	0	0	10.24	9.17	6.10	5.28	0.60
Non-POU domain-containing octamer-binding protein	55	2	2	2	0	3	2	2.10	0.59	1.25	1.08	0.60
Solute carrier organic anion transporter family member 1A1	74	3	3	7	2	4	3	3.08	1.31	1.85	0.42	0.60
Eukaryotic translation initiation factor 3 subunit H	40	2	1	4	2	1	1	2.83	1.79	1.70	0.71	0.60
Ester hydrolase C11orf54 homolog	35	0	2	2	1	3	0	2.37	2.21	1.44	1.44	0.61
Bile acid-CoA:amino acid N-acyltransferase	46	3	5	4	3	8	1	5.40	3.04	3.30	2.16	0.61
Myb-binding protein 1A	152	2	2	1	1	2	0	0.54	0.20	0.33	0.33	0.61
Proteasome subunit alpha type-2	26	12	6	8	5	7	5	16.96	4.78	10.47	1.02	0.62
Threonyl-tRNA synthetase, cytoplasmic	83	2	2	3	2	5	2	1.99	0.98	1.23	0.03	0.62
Metalloreductase STEAP4	53	3	3	4	2	3	1	3.08	0.46	1.91	0.93	0.62
1,4-alpha-glucan-branching enzyme	80	9	3	7	7	14	8	6.19	2.45	3.85	1.76	0.62
Protein transport protein Sec23B	86	1	2	2	0	2	1	0.95	0.34	0.59	0.59	0.62
Aspartyl aminopeptidase	52	1	2	2	0	2	1	1.58	0.56	0.98	0.97	0.62
Plasminogen activator inhibitor 1 RNA-binding protein	45	2	2	5	2	3	2	3.62	1.59	2.27	0.06	0.63
Peroxiredoxin-5, mitochondrial	22	9	5	11	6	0	1	14.60	12.80	9.18	5.98	0.63
Basigin	42	1	2	3	0	1	1	1.93	1.29	1.22	1.20	0.63
Glucokinase regulatory protein	65	5	3	6	4	7	4	4.55	0.88	2.89	0.49	0.63
L-serine dehydratase/L-threonine deaminase	35	15	7	14	8	12	10	19.15	1.68	12.20	2.57	0.64
Translationally-controlled tumor protein	19	5	4	4	2	4	2	11.20	1.43	7.15	2.99	0.64
Heme-binding protein 1	21	3	7	10	4	8	2	16.38	8.34	10.46	5.93	0.64
26S proteasome non-ATPase regulatory subunit 6	46	2	1	2	0	1	2	1.77	0.57	1.13	1.15	0.64
Galectin-9	40	2	1	2	0	1	2	2.03	0.66	1.30	1.32	0.64
Na(+)/H(+) exchange regulatory cofactor NHE-RF1	39	2	1	2	3	4	1	3.39	1.59	2.17	1.47	0.64
Biliverdin reductase A	34	1	1	3	0	1	2	2.38	1.59	1.53	1.55	0.64
Heat shock protein 105 kDa	96	1	1	3	0	1	2	0.84	0.56	0.54	0.55	0.64
Tubulin beta-2C chain	50	13	10	17	3	11	12	13.37	2.60	8.58	4.99	0.64
Pre-mRNA-processing-splicing factor 8	274	3	2	3	3	2	0	0.48	0.09	0.31	0.28	0.64
60S acidic ribosomal protein P2	12	5	5	8	3	0	0	17.38	16.13	11.18	10.56	0.64
Ras-related protein Rab-1A	23	11	10	7	4	14	6	22.95	8.25	14.79	6.64	0.64
Arylacetamide deacetylase	45	6	5	5	4	8	3	6.95	1.92	4.52	1.04	0.65

Fumarate hydratase, mitochondrial	54	26	9	15	17	18	11	17.90	5.13	11.65	3.80	0.65
Probable imidazolonepropionase	46	11	5	13	9	8	6	11.34	2.34	7.39	2.23	0.65
Coatomer subunit gamma	98	5	3	6	3	5	4	2.67	0.23	1.74	0.35	0.65
Cytochrome P450 4A12A	58	1	1	3	1	4	3	2.28	1.35	1.49	1.07	0.65
Homogentisate 1,2-dioxygenase	50	10	7	14	11	14	6	12.46	2.41	8.14	2.55	0.65
2-oxo-4-hydroxy-4-carboxy-5-ureido imidazoline decarboxylase	20	4	1	1	3	6	3	9.14	6.47	5.99	3.01	0.66
Ribonuclease inhibitor	50	3	2	2	0	3	3	2.63	0.63	1.73	1.60	0.66
Mitochondrial 2-oxoglutarate/malate carrier protein	34	6	4	8	6	8	4	10.61	1.79	7.00	1.63	0.66
Programmed cell death 6-interacting protein	96	4	4	4	4	9	3	2.93	1.60	1.95	0.26	0.66
Isopentenyl-diphosphate Delta-isomerase 1	26	1	2	0	0	2	0	1.93	1.96	1.29	2.24	0.67
Ras-related protein R-Ras	24	0	1	1	1	2	0	2.08	2.13	1.40	1.21	0.67
Nucleophosmin	33	3	3	7	3	4	3	6.92	2.93	4.65	0.13	0.67
Aflatoxin B1 aldehyde reductase member 2	41	6	2	5	4	3	3	5.56	1.71	3.74	1.23	0.67
Histone H1.2	21	2	2	7	3	5	4	10.90	5.75	7.34	2.62	0.67
Glutamyl aminopeptidase	108	3	2	0	2	3	0	0.92	0.80	0.62	0.54	0.67
Inter-alpha-trypsin inhibitor heavy chain H2	106	2	1	0	1	1	0	0.47	0.46	0.32	0.27	0.68
Poly(rC)-binding protein 2	38	2	2	0	0	1	0	1.30	1.28	0.88	1.53	0.68
PDZ and LIM domain protein 1	36	1	2	1	0	1	0	1.37	0.05	0.93	1.62	0.68
Vesicular integral-membrane protein VIP36	40	2	2	1	1	3	1	2.48	1.32	1.70	0.71	0.68
Eukaryotic translation initiation factor 3 subunit I	36	1	0	0	1	2	1	1.39	1.42	0.95	0.83	0.68
Secernin-3	48	1	0	0	1	2	1	1.05	1.06	0.71	0.62	0.68
Lysosome-associated membrane glycoprotein 2	46	1	1	0	0	2	1	1.09	1.11	0.75	0.65	0.68
Vimentin	54	1	1	0	0	2	1	0.93	0.94	0.64	0.55	0.68
Mitochondrial import inner membrane translocase subunit TIM44	51	1	1	0	0	2	1	0.98	1.00	0.67	0.58	0.68
Cation-dependent mannose-6-phosphate receptor	31	1	1	0	0	2	1	1.62	1.65	1.11	0.96	0.68
Ribose-phosphate pyrophosphokinase 1	35	1	2	2	1	3	1	2.83	1.49	1.94	0.81	0.69
Alanine--glyoxylate aminotransferase 2-like 1	55	0	0	2	2	1	0	0.89	0.87	0.61	1.06	0.69
Eukaryotic translation initiation factor 2A	64	0	1	2	1	1	0	0.76	0.75	0.52	0.45	0.69
Heterogeneous nuclear ribonucleoprotein L	64	0	1	2	1	1	0	0.76	0.75	0.52	0.45	0.69
THO complex subunit 4	27	0	2	2	0	1	0	1.81	1.77	1.24	2.15	0.69
Uncharacterized protein C18orf19 homolog	32	0	1	1	0	2	1	1.56	1.59	1.07	0.93	0.69

Trans-2-enoyl-CoA reductase, mitochondrial	40	0	1	1	0	2	1	1.25	1.28	0.86	0.74	0.69
Leukotriene A-4 hydrolase	69	3	4	3	1	3	1	2.14	0.07	1.47	1.25	0.69
EH domain-containing protein 3	61	3	3	2	1	1	0	1.60	0.78	1.10	1.26	0.69
Thioredoxin domain-containing protein 12	19	3	1	0	1	0	0	2.56	4.43	1.77	1.53	0.69
Trans-2,3-enoyl-CoA reductase	36	5	4	3	2	4	2	5.47	1.40	3.77	1.58	0.69
Actin-related protein 2/3 complex subunit 2	34	2	4	3	0	4	2	4.36	1.57	3.01	2.96	0.69
Myosin-11	227	3	2	0	0	0	0	0.21	0.37	0.15	0.26	0.69
Eukaryotic translation initiation factor 6	27	2	1	2	2	2	1	3.64	0.12	2.51	1.05	0.69
Elongation factor 1-delta	31	3	2	4	3	5	3	6.37	1.77	4.40	1.01	0.69
Poly(rC)-binding protein 1	37	3	3	4	2	5	3	5.33	1.48	3.69	0.85	0.69
Selenide, water dikinase 2	48	7	6	6	3	5	3	6.13	0.90	4.24	1.78	0.69
Signal transducer and activator of transcription 3	88	3	4	4	1	2	1	1.66	0.51	1.15	0.98	0.69
Heme oxygenase 1	33	2	2	1	0	0	0	1.47	1.47	1.02	1.76	0.69
Sedoheptulokinase	51	2	2	1	0	0	0	0.95	0.95	0.66	1.14	0.69
Ubiquitin-conjugating enzyme E2 L3	18	5	4	4	2	0	0	8.05	7.12	5.59	5.59	0.69
Fumarylacetoacetate hydrolase domain-containing protein 1	25	4	4	3	0	2	2	5.87	1.86	4.09	4.03	0.70
Brain protein 44	14	1	1	2	1	0	0	3.44	3.42	2.40	2.07	0.70
Legumain	49	1	2	2	0	0	0	0.98	0.98	0.69	1.19	0.70
Inorganic pyrophosphatase	33	17	10	18	13	20	14	27.35	3.12	19.14	3.59	0.70
Actin-related protein 2/3 complex subunit 3	21	1	0	3	2	2	2	4.67	2.26	3.27	2.83	0.70
2-amino-3-ketobutyrate coenzyme A ligase, mitochondrial	45	5	5	4	4	10	4	7.00	3.80	4.92	0.60	0.70
39S ribosomal protein L22, mitochondrial	24	0	1	2	0	1	1	2.04	1.99	1.43	1.24	0.70
Proteasome activator complex subunit 2	27	3	3	6	1	3	4	7.23	2.94	5.09	3.00	0.70
NADH-cytochrome b5 reductase 3	34	19	16	30	13	28	23	37.12	8.61	26.18	8.49	0.71
Glucose-6-phosphatase	40	1	1	3	0	2	3	2.45	1.19	1.74	2.02	0.71
ADP-ribosylation factor GTPase-activating protein 2	57	2	1	1	0	0	1	0.85	0.85	0.60	0.52	0.71
Clathrin interactor 1	69	2	1	1	0	0	1	0.70	0.71	0.50	0.43	0.71
39S ribosomal protein L12, mitochondrial	22	2	0	0	0	1	2	2.25	2.21	1.60	2.77	0.71
Heterogeneous nuclear ribonucleoproteins C1/C2	34	1	0	2	1	0	1	1.41	1.41	1.01	0.87	0.71
Propionyl-CoA carboxylase alpha chain, mitochondrial	80	7	5	9	8	10	5	5.34	1.06	3.82	1.04	0.72
2-hydroxyacyl-CoA lyase 1	64	8	6	8	3	3	4	4.82	2.10	3.46	1.19	0.72

Serine/threonine-protein phosphatase 2A catalytic subunit alpha isoform	36	0	0	2	0	1	2	1.36	1.33	0.98	1.69	0.72
Protein ETHE1, mitochondrial	28	3	3	2	2	5	2	5.91	2.91	4.25	1.00	0.72
60S ribosomal protein L14	24	5	2	4	4	4	3	8.87	1.13	6.39	2.10	0.72
Protein disulfide-isomerase A5	59	4	6	5	1	1	0	2.74	1.67	1.99	2.74	0.73
Thyroid hormone-inducible hepatic protein	17	2	1	3	2	5	4	9.72	4.77	7.09	4.83	0.73
Glyceraldehyde-3-phosphate dehydrogenase	36	62	39	77	58	72	51	96.01	10.59	70.05	13.79	0.73
GTP-binding protein SAR1b	22	6	6	8	7	10	4	17.94	4.98	13.10	3.25	0.73
Putative adenosylhomocysteinase 2	59	1	0	2	0	0	2	0.82	0.81	0.60	1.03	0.73
5'(3')-deoxyribonucleotidase, cytosolic type	23	4	2	4	3	2	2	7.07	2.28	5.17	1.20	0.73
Glycine N-acyltransferase-like protein Keg1	34	2	3	6	2	2	2	4.77	3.18	3.50	0.82	0.73
Ubiquitin	9	14	13	12	6	15	10	74.80	10.64	54.93	19.86	0.73
Serine hydroxymethyltransferase, cytosolic	53	5	5	11	4	4	5	6.12	3.31	4.50	0.63	0.74
Corticosteroid 11-beta-dehydrogenase isozyme 1	32	14	4	10	10	10	10	17.39	3.41	12.82	5.67	0.74
Glutathione S-transferase P 1	24	85	63	83	73	100	55	183.36	25.42	135.26	16.27	0.74
60S ribosomal protein L7	31	7	2	2	5	5	3	7.43	4.01	5.48	2.45	0.74
Cystathionine gamma-lyase	44	11	10	12	9	12	6	13.04	0.87	9.64	2.20	0.74
Putative ATP-dependent Clp protease proteolytic subunit, mitochondrial	30	2	1	2	2	3	2	3.84	1.09	2.85	1.02	0.74
Methionyl-tRNA synthetase, cytoplasmic	101	2	1	2	2	3	2	1.14	0.32	0.85	0.30	0.74
2-oxoisovalerate dehydrogenase subunit alpha, mitochondrial	50	3	0	4	2	3	5	3.27	0.49	2.43	2.66	0.74
Malate dehydrogenase, mitochondrial	36	22	20	30	18	25	17	34.99	5.07	25.99	1.72	0.74
T-complex protein 1 subunit gamma	61	3	4	4	4	4	0	2.96	0.50	2.20	1.90	0.74
Choline dehydrogenase, mitochondrial	66	9	5	5	7	4	1	4.45	1.91	3.31	2.31	0.75
Plastin-2	70	2	3	4	1	1	1	1.62	1.02	1.21	0.82	0.75
Heat shock protein HSP 90-alpha	85	9	9	12	8	15	9	6.97	1.93	5.21	0.44	0.75
Mimitin, mitochondrial	20	1	3	0	0	3	0	3.36	3.91	2.52	4.36	0.75
Succinate dehydrogenase [ubiquinone] iron-sulfur subunit, mitochondrial	32	4	5	11	6	10	7	12.82	5.84	9.61	1.84	0.75
Serotransferrin	77	10	3	7	7	8	8	5.32	0.99	4.00	1.82	0.75
Tubulin beta-5 chain	50	3	0	2	2	2	3	2.29	0.55	1.72	1.60	0.75

Voltage-dependent anion-selective channel protein 2	32	5	1	1	3	5	4	5.69	3.64	4.29	2.53	0.75
3 beta-hydroxysteroid dehydrogenase type 5	42	1	2	5	1	1	2	2.69	2.60	2.03	0.73	0.76
Bifunctional 3'-phosphoadenosine 5'-phosphosulfate synthase 2	70	2	1	0	2	2	0	0.95	0.82	0.72	0.72	0.76
Mitochondrial ornithine transporter 1	33	8	8	17	6	15	15	19.87	7.03	15.10	7.82	0.76
Sepiapterin reductase	28	11	10	10	6	9	6	17.53	1.42	13.35	4.02	0.76
AP-1 complex subunit sigma-1A	19	0	0	1	2	3	1	3.52	4.12	2.69	2.65	0.76
T-complex protein 1 subunit eta	60	3	2	6	3	2	3	2.97	1.61	2.28	0.52	0.77
Transcriptional activator protein Pur-beta	34	2	1	1	2	1	0	1.92	0.81	1.48	1.48	0.77
Complement component C8 gamma chain	23	2	0	2	4	4	2	5.75	2.70	4.44	4.37	0.77
Signal peptidase complex subunit 2	25	1	0	1	2	2	1	2.65	1.24	2.04	2.01	0.77
28S ribosomal protein S26, mitochondrial	23	1	1	1	1	2	1	2.88	1.35	2.22	0.06	0.77
Bifunctional UDP-N-acetylglucosamine 2-epimerase/N-acetylmannosamine kinase	79	1	1	1	1	2	1	0.84	0.39	0.65	0.02	0.77
Platelet-activating factor acetylhydrolase IB subunit beta	26	1	1	1	1	2	1	2.54	1.19	1.97	0.05	0.77
26S protease regulatory subunit 4	49	5	3	4	5	3	1	4.00	0.92	3.10	2.03	0.77
40S ribosomal protein S3a	30	6	8	7	4	7	3	10.93	1.10	8.47	4.36	0.77
Beta-lactamase-like protein 2	33	4	4	3	2	5	3	5.99	1.69	4.65	1.53	0.78
Ras-related protein Rab-18	23	2	1	3	3	7	5	8.66	6.02	6.73	4.64	0.78
40S ribosomal protein S14	16	5	4	3	2	0	0	8.06	7.64	6.29	6.29	0.78
Far upstream element-binding protein 2	77	2	1	1	1	1	1	0.85	0.36	0.66	0.02	0.78
F-actin-capping protein subunit beta	31	2	1	2	2	0	0	2.07	1.80	1.62	1.62	0.78
NADH dehydrogenase [ubiquinone] 1 beta subcomplex subunit 4	15	2	2	2	1	0	0	4.29	3.71	3.35	3.36	0.78
Calmodulin	17	5	4	7	5	0	0	11.33	10.18	8.88	7.83	0.78
Transthyretin	16	3	1	4	4	1	1	8.09	4.47	6.33	5.39	0.78
3-oxo-5-beta-steroid 4-dehydrogenase	37	3	2	2	1	3	3	3.55	0.85	2.78	1.46	0.78
Quinone oxidoreductase-like protein 2	38	3	2	3	2	2	2	3.43	0.65	2.69	0.07	0.78
Surfeit locus protein 1	35	1	1	1	0	2	2	1.89	0.89	1.48	1.51	0.78
Hydroxysteroid dehydrogenase-like protein 2	54	1	1	2	1	1	1	1.21	0.49	0.95	0.03	0.79
Cytochrome b5	15	12	10	12	6	0	2	25.72	22.28	20.23	13.27	0.79
UPF0480 protein C15orf24 homolog	26	0	0	2	1	2	2	2.53	2.20	2.00	2.03	0.79

Peroxisomal coenzyme A diphosphatase NUDT7	27	7	7	16	10	14	11	22.46	8.58	17.71	4.28	0.79
Selenium-binding protein 1	53	21	22	31	15	26	22	24.08	4.42	19.00	4.16	0.79
Proteasome subunit alpha type-7	28	8	6	8	10	14	7	17.68	6.76	13.97	3.64	0.79
UDP-glucuronosyltransferase 1-6	60	11	7	9	7	9	8	7.91	0.90	6.25	0.67	0.79
Cleavage and polyadenylation specificity factor subunit 5	26	6	2	2	2	0	2	4.97	5.72	3.93	0.11	0.79
Eukaryotic translation initiation factor 4H	27	3	2	1	3	5	2	5.54	3.84	4.41	1.02	0.80
Plastin-3	71	3	5	6	3	4	2	2.99	1.00	2.38	1.06	0.80
26S proteasome non-ATPase regulatory subunit 2	100	7	6	8	6	11	8	4.28	1.17	3.42	0.69	0.80
Agmatinase, mitochondrial	38	5	5	7	6	10	6	9.54	3.57	7.63	0.89	0.80
H-2 class I histocompatibility antigen, Q8 alpha chain	37	0	0	3	1	1	2	1.75	1.97	1.40	1.42	0.80
Isoamyl acetate-hydrolyzing esterase 1 homolog	28	4	4	6	3	3	3	7.55	2.45	6.07	0.98	0.80
B-cell receptor-associated protein 31	28	3	2	5	3	5	5	7.62	2.10	6.13	2.98	0.80
Cytochrome P450 2F2	56	12	10	7	6	8	5	7.89	2.29	6.36	2.30	0.81
SAM domain and HD domain-containing protein 1	73	1	1	1	3	3	0	1.14	0.83	0.92	1.05	0.81
Elongation factor 1-gamma	50	3	3	3	2	3	2	2.95	0.10	2.38	0.56	0.81
Protein canopy homolog 2	21	2	2	3	2	4	3	7.06	2.55	5.70	1.58	0.81
60 kDa heat shock protein, mitochondrial	61	48	51	65	42	55	38	45.07	6.38	36.51	4.92	0.81
Calnexin	67	4	4	7	4	7	6	4.41	1.31	3.58	0.99	0.81
Methylmalonyl-CoA mutase, mitochondrial	83	5	5	6	2	7	7	3.56	0.69	2.90	1.62	0.81
Eukaryotic translation initiation factor 5A-1	17	3	3	6	4	0	0	8.49	8.44	6.90	6.16	0.81
Alpha-mannosidase 2	132	2	2	1	2	2	0	0.62	0.23	0.51	0.44	0.81
Ubiquitin-conjugating enzyme E2 K	22	2	4	1	0	2	0	3.74	1.36	3.05	5.29	0.82
Phosphate carrier protein, mitochondrial	40	7	9	9	9	13	5	11.95	4.16	9.74	2.73	0.82
Vacuolar protein sorting-associated protein 13C	420	4	3	6	1	3	6	0.50	0.16	0.41	0.32	0.82
Cytochrome P450 2D10	57	4	4	8	3	6	7	5.16	1.66	4.22	2.00	0.82
Cytosol aminopeptidase	56	21	19	21	14	19	15	17.82	0.48	14.59	2.30	0.82
Nicotinate-nucleotide pyrophosphorylase [carboxylating]	32	4	9	10	4	10	6	12.32	5.42	10.11	3.94	0.82
Dolichyl-diphosphooligosaccharide--protein glycosyltransferase subunit 2	69	13	12	12	7	8	7	7.80	1.69	6.40	2.04	0.82

Carbamoyl-phosphate synthase [ammonia], mitochondrial	165	573	426	562	441	464	395	158.4	13.3	130.1	4.1	0.82
Translin	26	2	2	1	1	2	1	3.17	1.15	2.61	1.09	0.82
Coatomer subunit beta'	102	7	2	4	4	3	5	2.24	0.97	1.85	0.80	0.83
Translocon-associated protein subunit alpha	32	1	3	2	0	2	1	2.57	0.91	2.12	2.40	0.83
60S ribosomal protein L27a	17	1	2	3	2	1	0	4.77	3.18	3.95	3.42	0.83
60S ribosomal protein L32	16	4	3	1	1	0	0	5.05	6.34	4.19	4.81	0.83
Chaperone activity of bc1 complex-like, mitochondrial	72	2	4	5	2	3	2	2.27	0.99	1.89	0.79	0.83
5-hydroxyisourate hydrolase	14	3	2	2	2	0	0	5.75	5.30	4.79	4.15	0.83
NADH-ubiquinone oxidoreductase chain 1	36	2	1	2	2	1	1	2.26	0.73	1.88	0.79	0.83
Xylulose kinase	60	2	0	3	4	0	0	1.34	1.22	1.12	1.93	0.84
Superoxide dismutase [Cu-Zn]	16	14	7	11	12	0	1	25.15	22.31	21.00	17.22	0.84
NADH dehydrogenase [ubiquinone] 1 beta subcomplex subunit 10	21	15	12	9	7	17	14	32.17	10.63	26.88	9.33	0.84
Glycine N-acyltransferase	34	12	11	17	13	17	13	22.19	4.42	18.55	2.01	0.84
60S ribosomal protein L22	15	2	3	3	1	0	0	5.35	4.88	4.47	5.13	0.84
ADP-ribosylation factor 4	20	1	1	3	2	1	1	4.05	2.70	3.39	1.42	0.84
Non-specific lipid-transfer protein	59	51	39	54	38	42	41	40.70	3.91	34.09	2.24	0.84
40S ribosomal protein S3	27	14	12	13	18	27	14	33.08	15.55	27.73	5.57	0.84
14-3-3 protein beta/alpha	28	8	5	4	6	4	2	9.34	3.96	7.84	3.64	0.84
Methyltransferase-like protein 7B	28	12	12	13	6	11	11	21.03	1.10	17.68	6.00	0.84
Alpha-enolase	47	54	53	42	25	63	51	55.65	12.81	46.90	17.46	0.84
Sarcoplasmic/endoplasmic reticulum calcium ATPase 2	115	9	9	11	2	5	9	3.53	1.20	2.98	1.83	0.84
Thiosulfate sulfurtransferase	33	14	11	16	12	12	11	20.79	2.32	17.54	0.75	0.84
Tripeptidyl-peptidase 2	140	3	2	2	2	6	5	1.30	0.78	1.11	0.67	0.85
UTP--glucose-1-phosphate uridylyltransferase	57	13	13	11	7	20	16	12.74	4.56	10.82	4.35	0.85
GMP synthase [glutamine-hydrolyzing]	77	4	1	1	1	0	2	1.05	1.32	0.89	0.41	0.85
Catechol O-methyltransferase	29	20	16	14	13	17	13	28.84	5.31	24.64	2.77	0.85
Isochorismatase domain-containing protein 2A, mitochondrial	22	18	16	13	12	15	10	34.27	5.79	29.33	6.55	0.86
Alpha-aminoadipic semialdehyde synthase, mitochondrial	103	17	12	15	13	19	17	8.13	1.22	6.97	1.52	0.86
Acetyl-CoA acetyltransferase, cytosolic	41	8	7	10	9	11	8	11.61	2.09	9.97	1.25	0.86
Phenazine biosynthesis-like domain-containing protein2	32	3	1	6	5	2	3	5.57	3.02	4.79	3.14	0.86

Protein ERGIC-53	58	2	1	2	3	2	1	1.69	0.06	1.46	0.99	0.86
Rho GDP-dissociation inhibitor 2	23	9	5	4	7	5	3	12.81	5.56	11.04	4.21	0.86
Glycine N-methyltransferase	33	19	18	24	17	21	18	31.74	3.44	27.36	1.43	0.86
Monocarboxylate transporter 1	53	1	1	2	2	3	2	1.87	0.99	1.61	0.57	0.86
Malate dehydrogenase, cytoplasmic	37	17	12	19	19	23	18	26.20	4.88	22.59	5.42	0.86
Triosephosphate isomerase	27	14	11	18	14	15	14	28.48	3.33	24.63	3.62	0.87
Methylcrotonoyl-CoA carboxylase subunit alpha, mitochondrial	79	9	5	7	4	7	10	4.77	0.69	4.13	2.22	0.87
Hypoxanthine-guanine phosphoribosyltransferase	25	3	2	2	2	1	1	3.90	1.90	3.39	1.11	0.87
Prostaglandin reductase 2	38	2	1	3	3	1	1	2.56	1.22	2.23	1.51	0.87
PEST proteolytic signal-containing nuclear protein	19	0	0	1	0	5	5	5.31	7.13	4.62	8.01	0.87
Fructose-1,6-bisphosphatase 1	37	33	37	44	34	68	51	64.65	26.05	56.39	14.22	0.87
Putative L-aspartate dehydrogenase	30	12	10	14	13	18	14	24.12	5.78	21.05	3.95	0.87
Dihydropteridine reductase	26	9	6	14	9	13	15	22.69	5.07	19.80	9.62	0.87
Sodium/potassium-transporting ATPase subunit alpha-1	113	7	11	13	9	12	7	4.64	1.41	4.06	0.82	0.87
Argininosuccinate lyase	52	30	25	31	24	39	35	31.60	5.75	27.62	6.81	0.87
Voltage-dependent anion-selective channel protein 1	32	15	11	13	12	16	14	22.57	3.04	19.74	2.97	0.87
Staphylococcal nuclease domain-containing protein1	102	13	11	9	9	10	7	5.14	1.00	4.49	0.91	0.87
40S ribosomal protein S2	31	7	6	5	7	8	4	10.62	2.74	9.30	2.31	0.88
Ubiquitin carboxyl-terminal hydrolase isozyme L3	26	2	1	1	3	4	2	4.48	3.07	3.93	1.93	0.88
Pyruvate dehydrogenase E1 component subunit alpha, somatic form, mitochondrial	43	1	1	2	3	4	2	2.70	1.85	2.38	1.17	0.88
Hypoxia up-regulated protein 1	111	20	14	13	14	14	12	6.93	1.64	6.13	0.37	0.88
Dipeptidyl peptidase 4	87	5	1	4	8	5	3	2.64	0.39	2.34	2.07	0.89
Dihydrolipoyllysine-residue succinyltransferase component of 2-oxoglutarate dehydrogenase complex, mitochondrial	49	7	5	6	2	6	9	6.35	0.55	5.62	3.84	0.89
Glycogen phosphorylase, liver form	97	40	23	23	29	32	29	16.07	4.41	14.24	2.02	0.89
Aspartyl-tRNA synthetase, cytoplasmic	57	4	4	5	2	4	5	3.73	0.41	3.31	1.44	0.89
Acyl-coenzyme A synthetase ACSM1, mitochondrial	65	14	10	8	13	14	8	9.11	2.81	8.09	1.81	0.89

Serine/threonine-protein phosphatase 2A 65 kDa regulatory subunit A alpha isoform	65	1	2	3	2	3	2	1.77	0.89	1.57	0.04	0.89
Hemoglobin subunit alpha	15	20	17	23	18	4	5	50.59	32.32	44.98	23.80	0.89
Valacyclovir hydrolase	33	7	10	7	7	16	9	15.06	8.37	13.43	2.44	0.89
Phytanoyl-CoA dioxygenase, peroxisomal	39	3	1	2	3	2	2	2.94	0.70	2.62	1.29	0.89
Protein transport protein Sec61 subunit alpha isoform 1	52	3	2	2	2	2	2	2.20	0.53	1.97	0.05	0.89
Phytanoyl-CoA dioxygenase domain-containing protein 1	33	3	2	2	2	2	2	3.47	0.83	3.10	0.08	0.89
Phospholysine phosphohistidine inorganic pyrophosphate phosphatase	29	3	3	2	1	2	2	3.95	0.94	3.53	1.74	0.89
Phosphoglucomutase-1	62	11	14	14	4	9	11	8.94	1.72	7.99	4.23	0.89
40S ribosomal protein S6	29	0	3	4	0	3	3	3.96	3.49	3.55	3.08	0.90
Fructose-bisphosphate aldolase B	40	49	50	69	51	65	57	74.97	13.32	67.37	6.71	0.90
Pyridoxal kinase	35	2	0	3	3	2	3	3.26	0.73	2.94	2.55	0.90
Lipoamide acyltransferase component of branched-chain alpha-keto acid dehydrogenase complex, mitochondrial	53	2	1	4	3	1	2	2.14	1.35	1.93	0.95	0.90
Elongation factor 1-alpha 1	50	51	40	52	42	55	55	51.81	3.70	46.82	9.70	0.90
Early endosome antigen 1	161	7	6	4	5	5	3	1.63	0.47	1.47	0.45	0.90
Transgelin-2	22	2	1	2	3	4	3	6.01	2.82	5.44	2.74	0.91
Ferritin light chain 1	21	14	15	24	22	25	18	49.26	14.88	44.59	8.36	0.91
GTP-binding nuclear protein Ran	24	3	3	5	5	8	6	11.01	5.53	9.98	3.47	0.91
Cytochrome P450 2D26	57	6	7	4	5	7	3	4.91	1.46	4.46	1.71	0.91
Succinate dehydrogenase [ubiquinone] flavoprotein subunit, mitochondrial	73	7	7	10	4	5	8	4.90	1.54	4.45	1.55	0.91
Apoptosis-inducing factor 1, mitochondrial	67	5	5	10	15	12	4	6.63	2.79	6.05	4.51	0.91
ADP-ribosylation factor-like protein 1	20	2	1	3	3	3	3	6.56	1.49	5.99	3.01	0.91
Protein disulfide-isomerase A3	57	23	20	14	14	13	10	14.33	4.59	13.08	4.26	0.91
Liver carboxylesterase 31-like	63	2	2	2	3	5	3	2.37	1.45	2.17	0.50	0.91
Argininosuccinate synthase	47	98	84	98	81	85	82	97.78	4.90	89.51	2.69	0.92
Superoxide dismutase [Mn], mitochondrial	25	8	7	7	9	12	8	17.81	5.87	16.35	2.04	0.92
Regucalcin	33	26	18	38	34	28	29	45.55	8.60	41.85	12.75	0.92
Cytochrome P450 2C29	56	10	9	6	9	12	7	8.25	2.93	7.59	0.86	0.92
Dual specificity protein phosphatase 3	20	3	2	1	4	6	3	8.33	6.51	7.66	2.52	0.92
Collagen alpha-1(XIV) chain	193	2	3	3	2	4	3	0.77	0.28	0.71	0.16	0.92

Nitrilase homolog 1	36	3	2	4	5	2	1	4.07	1.24	3.75	2.88	0.92
Protein NipSnap homolog 1	33	7	5	3	6	9	6	9.52	4.83	8.78	1.02	0.92
Peptidyl-prolyl cis-trans isomerase A	18	11	15	15	8	0	0	23.19	20.71	21.43	20.99	0.92
Translocation protein SEC63 homolog	88	4	3	2	2	3	3	1.68	0.56	1.55	0.36	0.92
ADP-ribosylation factor 1	21	1	3	7	3	0	1	6.08	8.61	5.63	2.70	0.92
Sulfotransferase 1A1	34	10	9	9	9	9	7	13.49	0.83	12.49	1.42	0.93
Annexin A6	76	10	10	10	13	10	4	6.47	0.22	6.00	2.96	0.93
Electron transfer flavoprotein subunit alpha, mitochondrial	35	27	16	24	23	21	25	33.64	3.54	31.23	7.47	0.93
T-complex protein 1 subunit beta	57	2	2	5	4	2	2	2.56	1.41	2.38	0.99	0.93
60S ribosomal protein L17	21	2	3	4	4	4	2	7.82	2.77	7.26	2.28	0.93
Glutathione S-transferase Mu 2	26	5	4	2	1	2	3	5.65	3.21	5.25	2.99	0.93
NADH dehydrogenase [ubiquinone] iron-sulfur protein 2, mitochondrial	53	3	4	2	1	5	4	3.12	1.54	2.91	1.70	0.93
60S ribosomal protein L3	46	3	3	2	4	6	3	3.97	2.39	3.70	0.59	0.93
Abhydrolase domain-containing protein 14B	22	11	11	10	6	7	8	20.76	4.14	19.35	5.74	0.93
ES1 protein homolog, mitochondrial	28	9	9	11	5	7	10	15.72	3.02	14.66	5.10	0.93
Dehydrogenase/reductase SDR family member 4	28	4	5	7	6	9	7	11.76	4.72	10.98	2.10	0.93
Glutamine synthetase	42	6	5	6	8	9	6	8.24	2.33	7.70	1.79	0.93
Coatomer subunit beta	107	7	6	8	6	4	5	2.89	0.87	2.70	0.21	0.94
Fumarylacetoacetase	46	23	26	24	24	27	17	26.40	3.07	24.72	4.68	0.94
MOSC domain-containing protein 1, mitochondrial	38	4	1	7	8	9	9	8.67	3.48	8.13	5.97	0.94
Dihydrolipoyllysine-residue acetyltransferase component of pyruvate dehydrogenase complex, mitochondrial	68	3	2	4	6	4	2	2.65	0.45	2.49	1.69	0.94
60S ribosomal protein L18	22	3	4	4	2	3	3	7.43	1.11	6.97	2.30	0.94
Macrophage mannose receptor 1	165	3	4	4	2	3	3	0.99	0.15	0.93	0.31	0.94
Adenylate kinase 2, mitochondrial	26	9	9	16	12	15	15	25.23	7.26	23.67	6.50	0.94
Myosin-9	226	57	64	67	55	57	45	13.10	0.98	12.33	1.89	0.94
ADP/ATP translocase 2	33	25	27	47	37	43	40	57.13	17.58	53.80	11.55	0.94
Alcohol dehydrogenase 1	40	23	33	31	28	37	22	37.40	9.60	35.23	6.28	0.94
Thioredoxin-dependent peroxide reductase, mitochondrial	28	8	5	5	11	11	6	14.16	5.75	13.34	5.66	0.94
Arginase-1	35	52	53	61	49	47	43	74.69	7.72	70.45	5.76	0.94
SEC14-like protein 4	46	7	1	2	3	0	4	3.16	3.82	2.98	1.76	0.94
Complement C3	186	25	30	24	18	18	13	5.88	0.84	5.56	2.30	0.94
Glyoxalase domain-containing protein 4	33	3	3	3	4	6	4	6.01	2.82	5.69	0.97	0.95

Eukaryotic initiation factor 4A-I	46	10	11	11	11	14	10	12.51	2.64	11.84	0.33	0.95
40S ribosomal protein S10	19	6	5	10	6	4	7	17.09	7.37	16.18	3.09	0.95
Electron transfer flavoprotein subunit beta	28	29	30	30	21	29	29	51.47	1.23	48.74	9.57	0.95
NADH dehydrogenase [ubiquinone] flavoprotein 2, mitochondrial	27	5	3	3	5	4	3	7.29	1.86	6.92	2.07	0.95
AP-2 complex subunit alpha-2	104	4	2	3	3	4	5	1.74	0.31	1.65	0.80	0.95
Cytochrome b-c1 complex subunit Rieske, mitochondrial	29	5	11	11	6	8	5	13.53	4.90	12.86	5.44	0.95
PCTP-like protein	33	1	3	7	4	3	3	5.42	4.39	5.15	0.82	0.95
17-beta-hydroxysteroid dehydrogenase 13	34	4	3	4	3	3	4	5.28	0.68	5.03	1.02	0.95
Protein NipSnap homolog 3A	28	4	3	4	3	3	4	6.41	0.83	6.10	1.23	0.95
Proteasome subunit beta type-7	30	2	4	5	3	5	4	6.57	2.89	6.25	1.08	0.95
Fibrinogen gamma chain	49	4	7	1	2	10	5	5.12	4.82	4.87	2.61	0.95
Proteasome subunit alpha type-1	30	8	5	6	4	8	11	12.04	2.17	11.47	6.85	0.95
Alpha-methylacyl-CoA racemase	42	6	5	3	3	3	3	4.67	1.98	4.45	1.34	0.95
Estradiol 17-beta-dehydrogenase 12	35	4	5	3	7	9	3	7.59	4.84	7.25	2.77	0.96
Alpha-2-macroglobulin	166	18	22	17	14	17	12	5.13	0.20	4.91	1.54	0.96
ATP synthase subunit O, mitochondrial	23	20	22	21	20	24	18	46.39	5.94	44.37	3.51	0.96
Ras-related protein Rab-7a	23	5	6	4	3	4	3	9.25	1.18	8.85	3.71	0.96
Adenosine kinase	40	19	16	16	17	16	14	20.88	2.03	19.98	1.46	0.96
Indolethylamine N-methyltransferase	29	31	28	28	23	28	29	49.13	2.92	47.06	6.56	0.96
Bile acyl-CoA synthetase	76	8	8	8	4	8	10	5.17	0.17	4.96	2.16	0.96
Enoyl-CoA hydratase domain-containing protein 2, mitochondrial	32	5	4	4	6	5	3	7.18	1.06	6.89	2.30	0.96
ATP synthase subunit alpha, mitochondrial	60	60	65	50	35	60	57	46.49	5.87	44.64	13.46	0.96
10-formyltetrahydrofolate dehydrogenase	99	90	71	88	84	79	82	42.47	1.77	40.80	4.09	0.96
Vesicle-associated membrane protein-associated protein A	28	3	2	5	8	7	4	8.83	3.78	8.49	5.45	0.96
Dihydrolipoyl dehydrogenase, mitochondrial	54	9	10	10	10	9	6	8.49	0.37	8.16	2.00	0.96
Filamin-A	281	5	9	13	3	5	9	1.33	0.77	1.28	0.64	0.96
Dihydropyrimidine dehydrogenase [NADP+]	111	14	11	14	11	10	13	5.59	0.86	5.38	0.68	0.96
Transitional endoplasmic reticulum ATPase	89	24	22	23	17	18	21	11.93	1.45	11.49	1.64	0.96
Alcohol dehydrogenase [NADP+]	37	9	10	9	9	11	8	12.87	1.98	12.41	1.12	0.96

Serine protease inhibitor A3K	47	11	9	9	10	9	8	10.10	1.15	9.77	0.87	0.97
S-formylglutathione hydrolase	31	10	6	8	9	10	11	14.83	2.19	14.34	4.48	0.97
L-gulonolactone oxidase	50	4	5	6	4	5	5	4.91	0.94	4.77	0.66	0.97
Protein DJ-1	20	11	8	9	10	11	11	25.44	3.46	24.75	4.43	0.97
60S ribosomal protein L6	34	6	7	5	10	11	4	10.70	5.08	10.45	4.29	0.98
Polyadenylate-binding protein 1	71	5	5	7	5	3	4	3.43	1.28	3.35	0.33	0.98
Aldehyde dehydrogenase, mitochondrial	57	44	44	36	33	53	49	38.39	8.64	37.75	8.16	0.98
Hydroxyacylglutathione hydrolase, mitochondrial	34	11	8	4	9	7	4	10.62	5.06	10.45	3.75	0.98
SEC14-like protein 2	46	7	9	4	6	13	8	8.66	5.24	8.52	1.74	0.98
Sterol 26-hydroxylase, mitochondrial	61	4	3	6	4	7	9	4.58	1.33	4.52	2.85	0.99
Exportin-1	123	0	1	0	1	2	0	0.28	0.48	0.27	0.24	0.99
Proteasome subunit alpha type-5	26	4	5	8	5	6	7	11.32	3.63	11.18	2.61	0.99
Ornithine carbamoyltransferase, mitochondrial	40	34	41	34	27	36	31	42.63	2.85	42.12	8.91	0.99
NADH dehydrogenase [ubiquinone] iron-sulfur protein 3, mitochondrial	30	8	6	7	6	4	6	10.31	3.17	10.22	0.28	0.99
Carbonyl reductase [NADPH] 1	31	9	6	6	8	7	7	11.63	2.44	11.54	1.64	0.99
6-phosphogluconolactonase	27	5	3	8	5	4	8	10.24	3.48	10.17	5.06	0.99
Glutathione S-transferase A3	25	51	41	58	61	47	47	102.0	7.91	101.4	20.12	0.99
60S ribosomal protein L21	19	6	8	8	7	11	9	21.67	7.20	21.56	3.24	0.99
Mitochondrial import receptor subunit TOM70	68	1	1	0	3	3	0	0.99	1.15	0.99	1.13	1.00
78 kDa glucose-regulated protein	72	50	63	52	53	69	49	39.06	8.50	38.97	4.44	1.00
Peroxiredoxin-6	25	43	44	44	39	42	41	84.49	1.04	84.48	5.38	1.00
Protein transport protein Sec23A	86	8	8	8	7	10	10	4.97	0.83	4.97	1.05	1.00
L-lactate dehydrogenase A chain	36	38	35	35	34	31	31	47.25	3.78	47.26	1.80	1.00
Adenosylhomocysteinase	48	41	34	32	36	43	42	39.70	7.08	39.82	5.54	1.00
Phosphoenolpyruvate carboxykinase, cytosolic [GTP]	69	13	8	11	17	8	6	7.57	1.63	7.60	4.19	1.00
Phenylalanine-4-hydroxylase	52	9	9	13	14	9	7	9.73	1.93	9.78	3.35	1.00
Protein NDRG2	41	14	15	9	8	10	9	13.18	3.12	13.26	4.55	1.01
Titin	3906	0	1	1	2	2	0	0.01	0.01	0.01	0.01	1.01
DNA-(apurinic or apyrimidinic site) lyase	35	1	0	0	2	1	0	0.95	0.82	0.96	1.66	1.01
Cation transport regulator-like protein 2	20	0	0	0	1	2	1	1.70	2.94	1.72	1.49	1.01
Eukaryotic translation initiation factor 1A	17	0	0	0	1	2	1	2.00	3.46	2.02	1.75	1.01

Eukaryotic translation elon gation factor 1 epsilon-1	20	0	0	0	1	2	1	1.70	2.94	1.72	1.49	1.01
28S ribosomal protein S10, mitochondrial	19	0	0	0	1	2	1	1.79	3.10	1.81	1.57	1.01
Selenide, water dikinase 1	43	0	1	0	0	2	1	0.79	1.37	0.80	0.69	1.01
Zinc-binding alcohol dehydrogenase domain-containing protein 2	41	1	2	0	0	1	0	0.81	0.70	0.82	1.42	1.01
Cytochrome P450 2C70	56	1	2	0	0	1	0	0.59	0.51	0.60	1.04	1.01
MIF4G domain-containing protein	25	1	2	0	0	1	0	1.33	1.15	1.34	2.33	1.01
Catalase	60	38	41	36	31	37	36	30.31	1.44	30.66	4.30	1.01
3-hydroxyanthranilate 3,4-dioxygenase	33	22	22	29	25	20	22	35.12	6.03	35.59	2.29	1.01
Tetratricopeptide repeat protein 39C	65	0	1	1	2	3	1	1.03	1.20	1.04	0.44	1.01
Glutathione S-transferase A4	26	3	5	3	7	7	1	8.29	4.71	8.41	5.86	1.02
Talin-1	270	27	29	27	23	38	38	5.61	1.36	5.70	1.59	1.02
HIV Tat-specific factor 1 homolog	86	0	0	1	2	1	0	0.38	0.33	0.39	0.67	1.02
Adapter molecule crk	34	2	1	0	2	1	0	1.45	1.43	1.48	1.48	1.02
Mitochondrial import receptor subunit TOM22 homolog	16	1	2	2	6	10	5	13.63	15.86	13.87	6.67	1.02
Vitamin D-binding protein	54	1	1	0	1	2	1	0.93	0.94	0.95	0.03	1.02
Adenylyl cyclase-associated protein 1	52	1	2	0	0	2	1	0.97	0.98	0.98	0.97	1.02
3-ketoacyl-CoA thiolase, mitochondrial	42	71	57	55	56	57	66	71.35	9.98	72.71	8.76	1.02
Transcriptional activator protein Pur-alpha	35	3	2	0	3	4	2	3.33	3.00	3.40	0.79	1.02
Kinesin-1 heavy chain	110	2	2	0	1	2	1	0.60	0.52	0.62	0.26	1.02
Cullin-3	89	0	0	1	2	2	1	0.56	0.57	0.57	0.56	1.02
Heterogeneous nuclear ribonucleoprotein H	49	0	0	1	2	2	1	1.02	1.04	1.04	1.03	1.02
Protein disulfide-isomerase	57	46	55	45	36	36	34	36.40	3.72	37.25	9.82	1.02
Enoyl-CoA hydratase, mitochondrial	31	16	19	22	17	19	20	30.10	4.52	30.80	3.22	1.02
Actin-related protein 3	47	0	2	1	0	2	1	1.06	1.09	1.09	1.07	1.02
Ras-related protein Rab-5C	23	1	3	2	2	4	2	5.05	3.46	5.17	1.21	1.03
Peptidyl-tRNA hydrolase 2, mitochondrial	20	2	3	4	6	4	1	8.21	2.91	8.42	6.27	1.03
NADH dehydrogenase [ubiquinone] flavoprotein 1, mitochondrial	51	1	2	1	1	2	1	1.30	0.61	1.33	0.56	1.03
26S protease regulatory subunit 6B	47	1	3	3	3	2	0	2.09	1.01	2.14	1.85	1.03
Heterogeneous nuclear ribonucleoprotein M	78	3	2	2	5	4	2	1.90	0.69	1.95	1.10	1.03
Secernin-2	47	2	2	1	2	2	1	1.75	0.64	1.80	0.59	1.03
Cytochrome P450 3A13	57	2	3	1	1	2	1	1.44	0.53	1.49	1.01	1.03
UPF0465 protein C5orf33 homolog	51	2	4	1	0	2	1	1.61	0.59	1.66	2.05	1.03

Aminoacyl tRNA synthetase complex-interacting multifunctional protein 2	35	0	1	2	2	1	0	1.40	1.37	1.44	1.44	1.03
Peptidyl-prolyl cis-trans isomerase FKBP4	52	0	1	2	2	1	0	0.94	0.92	0.97	0.97	1.03
Septin-2	42	3	2	1	3	4	3	3.16	1.88	3.25	0.74	1.03
Transmembrane emp24 domain-containing protein 10	25	5	7	6	8	12	8	15.23	8.06	15.68	1.42	1.03
Major vault protein	96	6	3	5	11	6	3	2.91	0.37	2.99	2.40	1.03
Eukaryotic translation initiation factor 3 subunit B	91	5	4	1	3	2	1	1.44	1.11	1.48	0.83	1.03
3,2-trans-enoyl-CoA isomerase, mitochondrial	32	6	9	7	8	10	6	11.84	3.61	12.20	2.16	1.03
Ras GTPase-activating-like protein IQGAP1	189	4	7	4	3	3	1	0.95	0.12	0.98	0.81	1.03
Heat shock protein 75 kDa, mitochondrial	80	7	10	6	6	7	4	4.10	0.46	4.23	1.87	1.03
T-complex protein 1 subunit theta	60	2	4	2	1	2	1	1.64	0.05	1.69	1.44	1.03
OCIA domain-containing protein 1	28	3	4	2	2	4	3	5.30	1.94	5.48	1.81	1.03
Lysosome membrane protein 2	54	2	0	0	2	2	2	1.23	1.07	1.27	1.10	1.03
Ras-related protein Rab-10	23	1	0	0	1	2	2	2.18	2.22	2.26	2.29	1.03
40S ribosomal protein S25	14	3	1	0	2	0	0	3.47	6.02	3.59	3.59	1.03
V-type proton ATPase subunit E 1	26	2	1	0	1	1	1	1.90	1.87	1.97	0.05	1.03
Cytochrome c oxidase subunit 2	26	6	6	6	8	12	10	15.26	7.16	15.78	4.33	1.03
Ras suppressor protein 1	32	2	1	0	1	0	0	1.01	1.76	1.05	0.91	1.03
SWI/SNF complex subunit SMARCC2	133	2	1	0	1	0	0	0.24	0.42	0.25	0.22	1.03
Glycine dehydrogenase [decarboxylating], mitochondrial	113	3	3	3	4	2	1	1.15	0.22	1.19	0.67	1.03
Histone H2A type 2-A	14	3	2	0	1	0	0	3.47	6.02	3.59	3.60	1.03
Sodium-coupled neutral amino acid transporter 3	56	2	2	0	0	1	1	0.88	0.87	0.91	0.90	1.03
Methylmalonate-semialdehyde dehydrogenase [acylating], mitochondrial	58	26	30	22	25	22	15	19.76	1.87	20.44	6.30	1.03
60S ribosomal protein L28	16	2	2	0	0	0	0	2.03	3.51	2.10	3.63	1.04
Probable ATP-dependent RNA helicase DDX5	69	2	2	0	0	0	0	0.47	0.81	0.49	0.84	1.04
60S ribosomal protein L7a	30	7	8	4	4	7	6	9.87	3.04	10.22	3.37	1.04
60S ribosomal protein L5	34	3	6	7	6	7	5	8.21	3.41	8.50	0.65	1.04
Acyl-protein thioesterase 2	25	2	1	1	2	3	3	3.97	2.10	4.12	2.16	1.04
Ubiquitin-like modifier-activating enzyme 1	118	12	16	19	19	15	11	6.38	1.38	6.61	1.60	1.04
Long-chain specific acyl-CoA dehydrogenase, mitochondrial	48	7	7	7	8	6	5	6.81	0.39	7.07	1.46	1.04

Alanyl-tRNA synthetase, cytoplasmic	107	8	7	7	9	4	3	2.89	0.89	3.00	1.40	1.04
39S ribosomal protein L10, mitochondrial	29	2	2	1	1	1	1	2.25	0.95	2.34	0.98	1.04
LIM and SH3 domain protein 1	30	1	2	1	0	2	2	2.20	1.03	2.29	1.98	1.04
Peroxisomal 2,4-dienoyl-CoA reductase	31	6	5	5	6	6	6	9.00	1.14	9.35	1.09	1.04
Ribosome-recycling factor, mitochondrial	29	2	0	1	3	0	0	1.67	1.68	1.73	3.00	1.04
60S ribosomal protein L24	18	3	3	3	3	4	4	9.14	1.90	9.49	1.92	1.04
Tetratricopeptide repeat protein 38	52	0	0	1	1	2	2	0.96	0.98	1.00	1.01	1.04
Aldo-keto reductase family 1 member C13	37	12	13	11	10	9	9	14.13	1.69	14.70	2.62	1.04
Proteasome activator complex subunit 1	29	2	5	7	5	3	2	6.72	4.28	6.99	2.91	1.04
2-oxoglutarate dehydrogenase, mitochondrial	116	11	7	7	10	8	9	3.67	0.87	3.82	0.69	1.04
Aspartate aminotransferase, cytoplasmic	46	15	10	13	17	13	14	14.59	1.19	15.19	3.88	1.04
Nucleolin	77	4	5	4	3	2	2	2.11	0.68	2.20	0.97	1.04
Short-chain specific acyl-CoA dehydrogenase, mitochondrial	45	4	5	5	4	3	3	4.35	0.96	4.52	1.04	1.04
MOSC domain-containing protein 2, mitochondrial	38	9	8	8	8	8	9	10.77	0.73	11.22	1.09	1.04
Glutathione S-transferase Mu 1	26	46	44	52	50	43	47	88.67	6.09	92.37	6.24	1.04
Peptidyl-prolyl cis-trans isomerase F, mitochondrial	22	3	4	2	1	0	0	3.66	3.37	3.81	4.77	1.04
Acyl-protein thioesterase 1	25	6	6	4	3	5	6	9.84	2.04	10.25	3.67	1.04
Acyl-coenzyme A thioesterase 12	62	1	0	1	2	0	0	0.52	0.45	0.54	0.94	1.04
Constitutive coactivator of PPAR-gamma-like protein 1	122	0	2	3	1	3	3	0.81	0.70	0.84	0.44	1.04
Histidine triad nucleotide-binding protein 1	14	3	3	3	3	0	0	6.89	5.97	7.19	6.22	1.04
Adenylate kinase isoenzyme 4, mitochondrial	25	4	2	2	3	3	4	5.91	1.99	6.16	2.20	1.04
Cathepsin D	45	8	7	5	5	2	3	5.41	3.19	5.64	2.16	1.04
40S ribosomal protein S15	17	4	4	5	5	0	0	8.50	7.48	8.88	7.83	1.04
WD repeat-containing protein 1	66	1	2	1	0	0	0	0.49	0.42	0.51	0.88	1.04
Myosin light polypeptide 6	17	1	2	1	0	0	0	1.89	1.64	1.97	3.42	1.04
Magnesium-dependent phosphatase 1	19	1	2	1	0	0	0	1.69	1.47	1.77	3.06	1.04
Thiopurine S-methyltransferase	28	3	4	5	3	5	6	7.62	2.10	7.96	3.03	1.04
NADH dehydrogenase [ubiquinone] 1 alpha subcomplex subunit 12	17	1	0	2	3	0	0	2.83	2.81	2.96	5.12	1.04

Tropomyosin alpha-3 chain	33	2	4	5	2	5	6	5.97	2.63	6.24	3.27	1.05
Tubulin alpha-1C chain	50	13	9	15	14	14	19	13.76	0.96	14.39	5.49	1.05
Histidine triad nucleotide-binding protein 2, mitochondrial	17	1	2	2	1	0	0	2.83	2.81	2.96	2.96	1.05
Solute carrier family 2, facilitated glucose transporter member 2	57	3	2	3	3	2	3	2.29	0.43	2.39	0.55	1.05
Ras-related C3 botulinum toxin substrate 1	21	2	0	1	2	2	3	3.92	1.43	4.10	3.81	1.05
4-trimethylamino butyraldehyde dehydrogenase	54	4	5	5	3	2	3	3.31	1.30	3.46	1.04	1.05
Trans-1,2-dihydrobenzene-1,2-diol dehydrogenase	36	4	4	4	3	1	2	4.04	2.28	4.24	1.33	1.05
4-hydroxyphenylpyruvate dioxygenase	45	29	22	27	39	30	26	31.34	2.65	32.87	9.70	1.05
T-complex protein 1 subunit delta	58	3	1	1	2	0	1	1.11	1.28	1.17	0.49	1.05
Endoplasmic reticulum resident protein 29	29	3	2	1	1	0	1	2.23	2.57	2.34	0.98	1.05
Fibronectin	272	9	15	18	6	9	15	2.15	0.88	2.26	1.00	1.05
Tubulin beta-2A chain	50	3	3	1	0	0	1	1.29	1.49	1.36	1.53	1.05
25-hydroxycholesterol 7-alpha-hydroxylase	58	0	1	2	1	0	0	0.55	0.95	0.58	0.50	1.05
Peroxiredoxin-1	22	39	50	42	41	47	39	95.48	11.91	100.50	12.07	1.05
Cytoskeleton-associated protein 5	226	0	2	2	0	0	0	0.14	0.24	0.15	0.26	1.05
Mitochondrial glutamate carrier 1	35	5	4	6	4	2	5	6.02	2.76	6.34	1.03	1.05
Microsomal triglyceride transfer protein large subunit	99	24	21	19	22	27	28	11.62	2.37	12.25	2.31	1.05
Estradiol 17-beta-dehydrogenase 11	33	2	1	3	2	2	4	3.46	0.77	3.65	2.49	1.06
Translational activator of cytochrome c oxidase 1	32	1	0	1	1	1	2	1.54	0.05	1.62	1.65	1.06
Hepatoma-derived growth factor	26	1	0	1	1	1	2	1.89	0.06	2.00	2.03	1.06
26S proteasome non-ATP-ase regulatory subunit 7	37	2	1	2	1	2	4	2.66	0.09	2.81	2.50	1.06
Cell division control protein 42 homolog	21	4	2	3	2	2	5	6.98	2.21	7.38	4.48	1.06
Transketolase	68	20	20	18	20	18	17	13.49	0.83	14.25	0.94	1.06
1,5-anhydro-D-fructose reductase	34	2	2	1	0	0	1	1.42	1.43	1.50	1.48	1.06
Peroxisomal sarcosine oxidase	44	3	3	5	2	2	5	3.69	1.59	3.90	1.90	1.06
Calcium-binding protein p22	22	1	0	0	0	1	2	1.51	1.31	1.60	2.77	1.06
Phosphomannomutase 2	28	3	1	2	2	0	2	2.88	2.65	3.05	1.09	1.06
Malonyl-CoA decarboxylase, mitochondrial	55	0	0	2	1	1	2	0.89	0.87	0.94	0.96	1.06

Poly [ADP-ribose] polymerase 9	97	0	0	2	1	1	2	0.50	0.49	0.53	0.54	1.06
Mitochondrial antiviral-signaling protein	53	2	1	3	2	0	2	1.51	1.38	1.61	0.57	1.06
Phosphoglycerate kinase 1	45	17	24	23	23	31	26	25.98	8.48	27.66	2.48	1.06
Proteasome subunit alpha type-4	29	2	2	3	1	0	2	2.77	2.53	2.95	1.05	1.06
Urocanate hydratase	75	28	29	21	25	22	19	15.50	2.43	16.52	3.07	1.07
Murinoglobulin-2	162	3	3	5	1	1	5	0.90	0.58	0.96	0.66	1.07
Protein disulfide-isomerase A4	72	15	17	12	9	13	15	9.10	1.09	9.72	3.01	1.07
Succinate-semialdehyde dehydrogenase, mitochondrial	56	1	1	2	0	1	3	1.16	0.47	1.24	1.44	1.07
Heat shock cognate 71 kDa protein	71	37	51	30	34	45	31	25.96	6.08	27.75	7.31	1.07
Delta-1-pyrroline-5-carboxylate dehydrogenase, mitochondrial	62	25	24	27	24	26	32	20.61	0.90	22.05	4.46	1.07
Proline synthetase co-transcribed bacterial homolog protein	30	8	7	8	12	11	9	14.81	3.37	15.89	4.18	1.07
14-3-3 protein zeta/delta	28	7	7	5	10	11	7	13.58	5.88	14.57	2.94	1.07
Enoyl-CoA hydratase domain-containing protein 3, mitochondrial	32	4	9	10	11	9	4	11.79	4.95	12.67	5.50	1.08
ATP synthase subunit d, mitochondrial	19	17	17	16	18	22	22	47.61	10.03	51.22	8.57	1.08
Pyruvate kinase isozymes M1/M2	58	0	0	2	1	0	1	0.55	0.95	0.59	0.51	1.08
Proteasome subunit beta type-4	29	9	8	8	11	9	8	14.70	1.36	15.83	2.82	1.08
3-hydroxyisobutyrate dehydrogenase, mitochondrial	35	7	8	11	12	9	8	12.62	2.68	13.59	3.16	1.08
Histidine ammonia-lyase	72	14	17	12	16	20	15	10.53	3.23	11.34	0.48	1.08
Succinyl-CoA ligase [ADP-forming] subunit beta, mitochondrial	50	12	7	6	9	10	13	9.20	3.09	9.93	3.41	1.08
Sorbitol dehydrogenase	38	19	23	24	24	22	21	28.01	3.21	30.45	1.31	1.09
NADH-ubiquinone oxidoreductase 75 kDa subunit, mitochondrial	80	6	9	8	6	7	7	4.30	0.58	4.68	0.95	1.09
Aconitate hydratase, mitochondrial	85	19	24	18	28	31	20	13.20	4.69	14.39	2.08	1.09
Glyoxylate reductase/hydroxypyruvate reductase	35	7	10	14	7	6	11	12.53	5.73	13.67	3.31	1.09
Cysteine-rich protein 2	23	6	6	4	6	7	6	12.17	3.63	13.33	0.36	1.09
Bleomycin hydrolase	53	1	0	2	0	0	3	0.91	0.90	0.99	1.72	1.10
Dolichyl-diphosphooligosaccharide--protein glycosyltransferase subunit 1	69	11	16	16	12	11	12	8.99	1.83	9.86	1.59	1.10

Liver carboxylesterase 31	63	26	25	25	26	22	26	18.95	1.14	20.82	0.89	1.10
Succinyl-CoA ligase [GDP-forming] subunit alpha, mitochondrial	36	11	9	11	14	13	14	15.96	2.13	17.54	4.32	1.10
Kynurenine/alpha-aminoadipate amino-transferase, mitochondrial	48	5	8	4	4	6	4	5.14	1.20	5.66	2.37	1.10
Isocitrate dehydrogenase [NADP], mitochondrial	51	7	5	4	6	6	7	5.48	1.53	6.03	1.15	1.10
Heat shock 70 kDa protein 4	94	7	9	8	5	4	6	3.29	0.99	3.62	1.10	1.10
Tetratricopeptide repeat protein 36	20	7	6	3	6	5	4	12.31	4.94	13.57	2.63	1.10
Phosphoglycerate mutase 1	29	10	9	19	14	11	19	22.48	7.77	24.80	9.46	1.10
ATP synthase subunit beta, mitochondrial	56	79	88	71	66	74	84	65.54	4.30	72.46	11.46	1.11
14-3-3 protein gamma	28	6	4	6	8	4	5	9.32	1.76	10.32	3.67	1.11
Transcription factor BTF3 homolog 4	17	1	1	2	7	8	4	10.83	11.49	12.02	8.87	1.11
Carnitine O-palmitoyltransferase 2, mitochondrial	74	10	12	13	11	8	10	6.83	1.46	7.59	0.53	1.11
Glutamate dehydrogenase 1, mitochondrial	61	59	53	49	44	36	56	38.52	8.48	42.79	6.12	1.11
Peptide methionine sulfoxide reductase	26	12	11	9	12	7	7	17.58	4.45	19.56	4.75	1.11
ATP synthase subunit b, mitochondrial	29	22	25	21	16	17	23	33.81	3.58	37.64	8.62	1.11
Maleylacetoacetate isomerase	24	48	48	43	52	46	47	93.56	6.84	104.3	4.19	1.11
Retinol dehydrogenase 7	36	8	7	8	10	11	12	12.34	2.81	13.77	3.89	1.12
Carbonyl reductase family member 4	25	8	5	2	5	3	4	8.50	6.22	9.52	0.94	1.12
Medium-chain specific acyl-CoA dehydrogenase, mitochondrial	46	8	7	12	14	7	8	9.56	2.54	10.71	4.04	1.12
Isovaleryl-CoA dehydrogenase, mitochondrial	46	6	5	8	6	2	6	5.63	3.11	6.30	0.73	1.12
Major urinary protein 3	21	4	6	4	9	13	8	16.65	12.92	18.67	3.78	1.12
Amine oxidase [flavin-containing] B	59	7	5	7	5	2	7	4.39	2.31	4.93	1.15	1.12
Cytochrome c1, heme protein, mitochondrial	35	5	10	6	5	12	10	10.88	5.75	12.21	4.37	1.12
Eukaryotic translation initiation factor 3 subunit A	162	8	7	11	9	9	14	2.83	0.42	3.17	1.23	1.12
Membrane-associated progesterone receptor component 1	22	12	15	9	10	14	13	26.18	6.45	29.44	5.93	1.12
Trifunctional enzyme subunit beta, mitochondrial	51	20	19	9	16	16	14	14.50	5.47	16.33	2.20	1.13
Filamin-B	278	20	19	21	26	19	20	3.53	0.07	3.98	0.65	1.13
Aldehyde dehydrogenase X, mitochondrial	58	3	4	5	3	5	7	3.68	1.02	4.14	1.97	1.13

Mitochondrial inner membrane protein	84	7	8	7	8	8	8	4.30	0.48	4.87	0.13	1.13
Pyridoxine-5'-phosphate oxidase	30	2	4	4	5	4	2	5.47	1.94	6.20	2.47	1.13
Probable N-acetyltransferase CML2	26	8	7	9	10	6	8	14.43	2.44	16.37	2.89	1.13
Succinyl-CoA ligase [GDP-forming] subunit beta, mitochondrial	47	10	13	12	11	11	12	11.50	1.00	13.05	1.14	1.13
S-adenosylmethionine synthetase isoform type-1	44	11	17	10	13	18	13	14.63	5.43	16.62	2.48	1.14
60S ribosomal protein L10a	25	5	12	10	9	15	12	19.82	10.45	22.52	3.87	1.14
Epoxide hydrolase 2	63	27	36	37	31	35	41	25.75	4.25	29.26	4.80	1.14
Apolipoprotein O-like	29	3	5	1	2	5	3	5.16	3.57	5.87	2.63	1.14
Importin subunit beta-1	97	2	1	2	6	5	3	1.54	0.94	1.75	1.30	1.14
UDP-glucuronosyltransferase 1-1	60	5	2	5	6	2	5	3.25	1.34	3.70	1.78	1.14
14-3-3 protein epsilon	29	13	16	16	16	13	14	23.68	2.40	26.98	1.34	1.14
14-3-3 protein theta	28	3	2	3	5	4	4	5.87	1.22	6.70	2.78	1.14
Dimethylaniline monooxygenase [N-oxide-forming] 5	60	10	9	14	15	8	11	8.69	2.24	9.93	2.52	1.14
Alcohol dehydrogenase class-3	40	6	7	5	7	8	7	7.82	2.16	8.94	0.24	1.14
Peroxiredoxin-2	22	5	6	10	11	5	5	14.79	6.02	16.94	7.14	1.15
Estradiol 17 beta-dehydrogenase 5	37	41	38	43	44	38	52	53.95	1.60	61.84	11.32	1.15
Quinone oxidoreductase	35	5	6	8	10	6	5	8.87	1.99	10.17	3.67	1.15
Eukaryotic translation initiation factor 3 subunit M	43	4	5	3	2	3	4	3.81	0.63	4.36	1.82	1.15
60S acidic ribosomal protein P0	34	15	16	14	14	10	13	18.72	3.34	21.51	1.91	1.15
Eukaryotic translation initiation factor 5B	138	4	7	5	7	8	5	2.03	0.82	2.34	0.37	1.15
Ubiquitin thioesterase OTUB1	31	5	6	1	3	2	0	4.23	3.25	4.87	4.87	1.15
UDP-glucuronosyltransferase 2B5	61	6	6	9	6	5	10	5.34	1.52	6.18	2.13	1.16
NAD(P) transhydrogenase, mitochondrial	114	10	10	8	10	8	9	3.73	0.48	4.33	0.15	1.16
ATP-binding cassette sub-family A member 6	183	8	10	13	10	7	11	2.49	0.79	2.89	0.24	1.16
Nucleoside diphosphate kinase A	17	6	3	3	6	0	1	8.54	8.58	9.91	7.37	1.16
ATP-dependent RNA helicase DDX1	83	2	3	4	3	3	4	1.77	0.57	2.06	0.42	1.16
3'(2'),5'-bisphosphate nucleotidase 1	33	3	4	4	3	2	3	4.44	1.35	5.15	0.83	1.16
ATP synthase subunit gamma, mitochondrial	33	11	13	12	15	10	9	16.35	0.98	19.02	4.29	1.16
Very long-chain acyl-CoA synthetase	70	20	25	25	24	15	18	13.97	3.08	16.25	2.37	1.16

Dimethylglycine dehydrogenase, mitochondrial	97	31	32	30	29	33	44	15.89	1.31	18.51	4.73	1.16
Heterogeneous nuclear ribonucleoprotein K	51	4	4	5	7	7	7	5.17	1.64	6.03	1.81	1.17
Alpha-1-antitrypsin 1-3	46	7	8	9	9	9	11	8.91	1.34	10.40	1.98	1.17
NADH dehydrogenase [ubiquinone] 1 alpha subcomplex subunit 8	20	4	5	6	6	6	7	13.12	2.98	15.37	2.94	1.17
3 beta-hydroxysteroid dehydrogenase/Delta 5-->4-isomerase type 3	42	2	1	1	4	4	3	2.77	1.90	3.25	1.85	1.17
Melanoma inhibitory activity protein 3	214	5	7	9	6	3	6	1.29	0.66	1.51	0.12	1.17
Heterogeneous nuclear ribonucleoproteins A2/B1	37	7	3	6	11	10	12	10.25	3.15	12.04	6.97	1.17
Polypyrimidine tract-binding protein 1	56	4	4	3	3	1	2	2.32	1.30	2.72	0.86	1.18
Lactoylglutathione lyase	21	19	19	20	23	22	27	47.65	5.05	56.11	11.13	1.18
Sideroflexin-2	36	3	1	2	3	3	5	3.65	0.87	4.30	2.96	1.18
Glutathione S-transferase omega-1	27	2	3	4	3	2	3	4.82	1.96	5.68	0.15	1.18
S-methyl-5'-thioadenosine phosphorylase	31	3	2	3	3	2	4	4.21	0.80	4.97	1.78	1.18
Receptor expression-enhancing protein 6	22	2	5	3	2	2	1	5.19	1.15	6.14	4.72	1.18
Catenin beta-1	85	4	1	1	5	2	2	1.35	0.87	1.60	1.22	1.18
Mitochondrial carrier homolog 2	33	4	2	3	4	1	3	3.93	2.21	4.64	1.53	1.18
Citrate synthase, mitochondrial	52	5	7	5	3	5	7	4.73	0.16	5.59	2.34	1.18
Tryptophan 2,3-dioxygenase	48	2	2	6	3	1	5	3.02	2.60	3.58	1.74	1.18
Glutathione S-transferase Mu 7	26	2	2	4	3	2	4	5.01	2.04	5.93	2.12	1.18
Aspartate aminotransferase, mitochondrial	47	24	29	19	21	21	23	22.32	2.78	26.44	4.34	1.18
Proteasome subunit beta type-3	23	2	5	7	4	6	8	10.69	5.63	12.67	5.02	1.18
26S proteasome non-ATPase regulatory subunit 1	106	4	3	2	1	2	5	1.23	0.52	1.46	1.01	1.19
C-1-tetrahydrofolate synthase, cytoplasmic	101	20	23	18	23	18	18	9.08	0.56	10.77	1.19	1.19
Uricase	35	27	22	22	29	23	31	33.70	3.74	39.99	7.65	1.19
Microtubule-associated protein 4	117	10	11	7	12	9	7	3.65	0.69	4.35	1.06	1.19
Dolichyl-diphosphooligosaccharide--protein glycosyltransferase 48 kDa subunit	49	2	4	4	3	1	1	2.31	1.46	2.75	1.54	1.19
ADP-ribosylation factor 6	20	5	4	2	3	0	1	5.65	6.13	6.75	3.78	1.19
Dynamin-like 120 kDa protein, mitochondrial	111	2	2	1	3	3	2	0.90	0.47	1.07	0.25	1.20

Fatty-acid amide hydrolase 1	63	2	4	1	1	3	2	1.58	0.83	1.89	1.21	1.20
Prohibitin-2	33	14	10	12	16	8	13	16.79	4.17	20.13	4.59	1.20
ATP-dependent RNA helicase DDX3X	73	3	1	2	3	2	4	1.57	0.38	1.88	1.11	1.20
Stress-70 protein, mitochondrial	74	31	31	24	37	34	35	19.77	3.99	23.71	2.27	1.20
Serum albumin	69	27	26	31	37	21	28	18.68	3.00	22.43	4.09	1.20
Delta-aminolevulinic acid dehydratase	36	5	5	5	10	8	6	8.24	2.67	9.91	3.62	1.20
Phenazine biosynthesis-like domain-containing protein 1	32	10	14	17	16	11	14	19.38	5.35	23.40	1.58	1.21
Pyruvate carboxylase, mitochondrial	130	63	71	52	67	49	53	20.64	2.54	24.96	3.12	1.21
UMP-CMP kinase	22	5	8	9	6	5	8	14.07	4.76	17.06	2.94	1.21
GTP:AMP phosphotransferase mitochondrial	25	11	13	11	13	8	9	19.59	2.84	23.76	4.15	1.21
Bifunctional coenzyme A synthase	62	2	2	3	4	1	1	1.57	0.75	1.91	1.22	1.21
Peroxisomal 3,2-trans-enoyl-CoA isomerase	43	2	3	3	3	1	1	2.26	1.08	2.75	1.32	1.22
40S ribosomal protein S17	16	3	5	3	2	0	0	6.03	5.22	7.34	7.92	1.22
Apolipoprotein A-I-binding protein	31	6	5	3	4	3	5	6.33	2.68	7.70	1.07	1.22
Eukaryotic translation initiation factor 3 subunit E	52	1	0	1	5	3	1	1.60	1.16	1.95	2.55	1.22
Elongation factor Tu, mitochondrial	50	5	6	4	3	3	5	3.92	0.91	4.78	1.57	1.22
60S ribosomal protein L18a	21	0	0	3	3	3	4	4.70	4.08	5.74	5.17	1.22
Nuclease-sensitive element-binding protein 1	36	3	3	2	2	1	2	2.71	1.32	3.31	0.77	1.22
2,4-dienoyl-CoA reductase, mitochondrial	36	16	21	26	19	18	30	27.22	6.63	33.27	9.33	1.22
Xanthine dehydrogenase/oxidase	147	6	9	9	5	3	7	1.98	0.94	2.43	0.69	1.23
Hydroxyacid oxidase 1	41	2	2	1	3	2	1	2.01	0.73	2.47	1.20	1.23
High mobility group protein B1	25	0	2	4	1	2	4	3.91	3.83	4.82	3.28	1.23
Methylcrotonoyl-CoA carboxylase beta chain, mitochondrial	61	5	3	3	5	3	5	2.95	0.90	3.64	1.01	1.23
Purine nucleoside phosphorylase	32	7	14	12	9	8	9	13.77	3.75	16.99	4.37	1.23
Aldehyde dehydrogenase family 8 member A1	54	14	7	5	12	8	13	8.20	4.13	10.13	3.21	1.24
Dipeptidyl peptidase 3	83	1	0	1	3	3	3	1.00	0.73	1.24	1.08	1.24
Vinculin	117	8	9	8	10	5	6	2.92	0.65	3.62	0.83	1.24
Selenium-binding protein 2	53	0	1	3	4	2	1	1.54	1.39	1.91	1.63	1.24
Integrin alpha-1	131	1	2	1	1	3	3	0.63	0.46	0.79	0.41	1.24
Bifunctional aminoacyl-tRNA synthetase	170	13	15	12	11	12	18	3.56	0.17	4.42	1.16	1.24

Peroxisomal acyl-coenzyme A oxidase 1	75	36	37	32	46	44	51	24.55	4.87	30.50	5.50	1.24
Leucine-rich PPR motif-containing protein, mitochondrial	157	6	6	8	9	7	10	2.19	0.30	2.72	0.73	1.24
Translocon-associated protein subunit delta	19	1	3	4	4	5	5	8.68	5.56	10.80	2.96	1.24
Prostaglandin E synthase 3	19	1	1	2	3	2	2	4.32	1.53	5.38	2.65	1.24
Histone H2B type 1-C/E/G	14	4	5	1	1	0	0	5.77	7.24	7.19	9.52	1.25
Aldose 1-epimerase	38	4	3	3	5	3	4	4.31	0.72	5.38	1.33	1.25
Peroxisomal bifunctional enzyme	78	15	25	22	18	23	29	12.63	2.94	15.78	4.01	1.25
Guanine nucleotide-binding protein subunit beta-2-like 1	35	7	7	4	5	4	6	7.01	2.36	8.76	1.46	1.25
Haloacid dehalogenase-like hydrolase domain-containing protein 2	29	2	1	1	2	2	3	2.84	1.03	3.55	1.86	1.25
Ubiquinone biosynthesis protein COQ9, mitochondrial	35	2	3	1	0	2	3	2.35	0.86	2.94	2.55	1.25
UDP-glucuronosyltransferase 3A2	60	3	2	1	2	1	2	1.36	0.93	1.70	0.05	1.25
Proliferation-associated protein 2G4	44	0	2	2	0	3	4	1.88	1.76	2.36	2.40	1.25
Formimidoyltransferase-cyclodeaminase	59	18	20	12	13	14	20	12.22	2.56	15.33	3.71	1.25
Heat shock protein HSP 90-beta	83	32	39	32	43	31	33	18.74	0.30	23.55	2.54	1.26
Omega-amidase NIT2	31	7	4	4	9	8	10	10.10	3.58	12.69	5.50	1.26
Proteasome subunit beta type-5	29	1	1	5	5	4	6	5.65	3.49	7.10	4.78	1.26
Murinoglobulin-1	165	20	26	16	19	16	18	5.16	0.66	6.49	1.25	1.26
Heterogeneous nuclear ribonucleoprotein A3	40	2	5	6	6	6	6	5.75	2.88	7.25	0.84	1.26
UDP-glucose:glycoprotein glucosyltransferase 1	176	8	12	14	16	11	12	3.07	0.80	3.86	0.61	1.26
40S ribosomal protein S9	23	5	3	4	7	5	7	9.99	1.47	12.63	5.27	1.26
Alpha-tocopherol transfer protein	32	10	12	10	9	8	13	14.30	1.35	18.14	3.69	1.27
Ribosome-binding protein1	173	21	32	25	23	18	23	6.04	0.81	7.67	1.44	1.27
Calcium-binding mitochondrial carrier protein Aralar2	74	19	25	23	35	24	21	14.63	2.03	18.58	4.61	1.27
Peptidyl-prolyl cis-trans isomerase B	24	7	10	11	15	13	13	21.24	6.74	26.98	5.39	1.27
Glutathione S-transferase kappa 1	26	15	12	9	16	12	16	22.72	5.80	28.86	4.93	1.27
Bile salt export pump	147	11	23	15	12	14	14	4.46	0.71	5.67	1.97	1.27
Canalicular multispecific organic anion transporter 1	174	10	10	7	8	6	10	2.16	0.56	2.74	0.38	1.27
Calcium-binding mitochondrial carrier protein Aralar1	75	0	1	2	4	2	0	0.88	0.76	1.12	1.39	1.27

Spectrin alpha chain, brain	285	21	25	22	21	8	16	2.90	1.28	3.69	0.73	1.27
Cytochrome b-c1 complex subunit 2, mitochondrial	48	6	10	9	3	4	10	6.43	2.38	8.20	4.39	1.28
COP9 signalosome complex subunit 8	23	0	1	1	2	3	2	2.91	3.40	3.71	1.32	1.28
Alpha-actinin-4	105	9	18	13	15	16	14	5.95	1.80	7.61	0.89	1.28
Estradiol 17-beta-dehydrogenase 8	27	1	3	1	1	2	1	2.45	1.15	3.14	2.13	1.28
Ribosome maturation protein SBDS	29	1	3	1	1	2	1	2.28	1.07	2.92	1.98	1.28
Kynurenine--oxoglutarate transaminase 1	48	2	2	1	3	1	0	1.36	0.58	1.75	1.60	1.28
Vesicle-trafficking protein SEC22b	25	3	6	3	5	6	4	7.94	3.72	10.19	1.83	1.28
Apolipoprotein E	36	3	7	8	7	2	2	5.84	4.19	7.50	3.96	1.28
Transmembrane emp24 domain-containing protein 9	27	0	1	2	3	2	1	2.44	2.12	3.13	2.12	1.28
40S ribosomal protein S4, X isoform	30	9	7	10	14	7	11	14.14	2.06	18.17	5.94	1.29
Inorganic pyrophosphatase 2, mitochondrial	38	2	2	0	1	2	2	1.75	1.52	2.25	0.80	1.29
Sarcosine dehydrogenase, mitochondrial	102	27	45	31	36	33	32	14.63	1.81	18.83	2.99	1.29
Prohibitin	30	15	18	18	22	17	22	27.30	2.58	35.24	4.44	1.29
Synaptosomal-associated protein 23	23	1	1	1	2	2	2	2.88	1.35	3.71	1.32	1.29
Parathymosin	11	1	1	1	2	2	2	6.01	2.82	7.77	2.77	1.29
Isocitrate dehydrogenase [NADP] cytoplasmic	47	14	19	13	16	18	21	15.75	3.33	20.34	3.22	1.29
Vacuolar protein sorting-associated protein 29	20	1	2	1	1	2	2	3.31	1.55	4.27	1.53	1.29
Peroxisomal membrane protein 2	22	3	3	2	4	3	3	5.98	1.42	7.73	1.23	1.29
Short/branched chain specific acyl-CoA dehydrogenase, mitochondrial	48	2	1	1	3	1	1	1.36	0.58	1.76	1.19	1.29
Phosphatidylethanolamine-binding protein 1	21	8	11	13	13	8	12	22.52	6.15	29.20	2.50	1.30
Splicing factor, arginine/serine-rich 3	19	0	0	1	1	3	4	3.52	4.12	4.58	5.79	1.30
Aspartyl/asparaginyl beta-hydroxylase	83	1	2	2	2	1	1	0.78	0.32	1.02	0.33	1.30
Hydroxyacyl-coenzyme A dehydrogenase, mitochondrial	34	15	22	20	20	17	23	25.03	3.36	32.60	3.04	1.30
Calreticulin	48	14	21	14	13	8	11	12.21	3.22	15.90	5.36	1.30
Cytochrome b5 type B	16	6	8	3	2	3	5	12.26	5.19	15.97	9.46	1.30
Phenylalanyl-tRNA synthetase alpha chain	58	2	2	2	3	0	0	1.11	0.96	1.45	1.32	1.30
Delta(3,5)-Delta(2,4)-dienoyl-CoA isomerase, mitochondrial	36	5	6	5	5	2	4	5.41	2.23	7.08	1.27	1.31

Electron transfer flavoprotein-ubiquinone oxidoreductase, mitochondrial	68	13	14	11	15	11	15	8.43	0.80	11.03	0.63	1.31
Propionyl-CoA carboxylase beta chain, mitochondrial	58	4	3	3	8	4	3	3.11	0.56	4.09	2.46	1.31
Coatomer subunit delta	57	1	1	2	2	1	2	1.14	0.46	1.50	0.53	1.31
GTP-binding protein SAR1a	22	1	2	2	1	1	2	2.96	1.20	3.88	1.39	1.31
F-actin-capping protein subunit alpha-2	33	3	2	4	5	1	3	3.92	2.17	5.15	2.30	1.31
60S ribosomal protein L9	22	10	11	7	13	12	13	21.71	6.31	28.67	3.11	1.32
C-Jun-amino-terminal kinase-interacting protein 4	146	1	3	2	3	4	3	0.80	0.55	1.05	0.03	1.32
Uncharacterized protein C10orf58 homolog	24	1	2	3	2	0	1	2.67	3.04	3.53	1.15	1.32
Dehydrogenase/reductase SDR family member 1	34	6	5	9	8	1	7	7.58	5.65	10.03	2.32	1.32
40S ribosomal protein S8	24	8	9	5	12	8	6	14.39	3.86	19.06	5.98	1.32
Endoplasmin	92	35	42	23	35	25	29	14.77	3.37	19.57	3.21	1.32
Peroxisomal multifunctional enzyme type 2	79	13	17	20	19	19	30	10.79	2.41	14.31	4.98	1.33
Keratin, type II cytoskeletal 5	62	6	4	3	6	2	4	2.89	1.61	3.84	0.89	1.33
Hydroxymethylglutaryl-CoA synthase, mitochondrial	57	53	78	50	46	44	64	42.18	2.94	56.21	14.40	1.33
Acyl-CoA synthetase family member 2, mitochondrial	68	8	15	14	11	7	11	6.94	2.53	9.25	1.62	1.33
Acyl-CoA-binding domain-containing protein 5	57	1	1	0	3	2	0	0.88	0.89	1.18	1.35	1.34
Ribosyldihydronicotinamide dehydrogenase [quinone]	26	6	11	5	6	6	5	10.73	1.36	14.34	6.07	1.34
Glutaminase liver isoform, mitochondrial	66	6	9	5	5	3	4	3.45	1.06	4.62	1.97	1.34
Myosin-XVIIIa	233	2	5	3	4	5	4	0.71	0.35	0.95	0.12	1.34
Cytoplasmic aconitate hydratase	98	26	26	16	31	26	31	11.41	3.14	15.31	1.73	1.34
Mitochondrial dicarboxylate carrier	32	6	7	5	5	3	6	7.12	2.19	9.58	1.60	1.35
Vacuolar protein sorting-associated protein 35	92	3	1	2	4	2	4	1.24	0.30	1.67	0.98	1.35
Flavin reductase	22	11	13	8	12	8	10	20.08	3.72	27.04	2.92	1.35
60S ribosomal protein L23a	18	2	7	12	10	10	14	21.87	14.37	29.49	10.79	1.35
Cytochrome P450 1A2	58	4	4	3	7	6	6	3.70	1.43	5.00	1.35	1.35
Actin, cytoplasmic 1	42	36	48	35	48	29	34	38.92	3.34	52.54	8.56	1.35
Spectrin beta chain, brain 1	274	12	21	13	7	6	12	1.84	0.63	2.48	1.30	1.35
Acyl-CoA dehydrogenase family member 11	87	4	5	3	4	3	4	1.88	0.31	2.54	0.31	1.35
Afadin	207	1	5	5	3	4	5	0.79	0.49	1.07	0.30	1.35

Proline dehydrogenase, mitochondrial	57	5	3	2	4	3	6	2.88	1.31	3.91	1.49	1.36
Proteasome subunit beta type-8	30	0	2	2	4	4	2	3.33	3.40	4.52	1.89	1.36
Abhydrolase domain-containing protein 11	34	0	2	1	1	2	1	1.47	1.50	2.00	0.84	1.36
Voltage-dependent anion-selective channel protein 3	31	2	3	5	5	3	5	5.26	2.30	7.16	1.99	1.36
Glutathione S-transferase theta-2	28	3	6	5	3	2	4	5.80	2.50	7.90	2.72	1.36
ATP-binding cassette sub-family F member 1	95	1	2	0	0	2	2	0.53	0.54	0.72	0.63	1.37
AP-2 complex subunit beta	105	3	4	1	3	2	1	0.94	0.47	1.29	0.72	1.37
Regulator of microtubule dynamics protein 1	35	3	5	1	2	2	1	2.82	1.40	3.86	2.97	1.37
Ethanolamine-phosphate cytidylyltransferase	45	1	4	2	2	3	2	2.20	1.16	3.02	1.26	1.37
Leucine-rich repeat-containing protein 59	35	5	6	7	6	4	9	7.45	1.92	10.27	2.84	1.38
Trifunctional enzyme subunit alpha, mitochondrial	83	32	41	24	37	35	43	18.03	3.89	24.86	2.45	1.38
Prenylcysteine oxidase	56	1	2	1	1	1	1	0.88	0.03	1.21	0.51	1.38
CCR4-NOT transcription complex subunit 1	267	1	2	1	1	1	1	0.18	0.01	0.25	0.11	1.38
Proteasome subunit alpha type-6	27	0	2	3	3	3	3	3.66	3.17	5.06	1.16	1.38
Ubiquitin carboxyl-terminal hydrolase 24	294	1	3	1	0	1	1	0.17	0.01	0.23	0.26	1.38
Clathrin heavy chain 1	192	43	57	35	48	37	48	9.81	1.09	13.56	1.24	1.38
Citrate lyase subunit beta-like protein, mitochondrial	38	3	2	2	5	1	1	2.57	1.25	3.55	2.73	1.38
Glutathione peroxidase 1	22	28	34	21	32	29	38	58.25	11.24	80.64	9.29	1.38
UPF0317 protein C14orf159 homolog, mitochondrial	66	2	1	1	3	0	0	0.73	0.74	1.02	1.16	1.39
Neutral alpha-glucosidase AB	107	10	12	10	15	7	9	4.12	0.68	5.71	1.31	1.39
26S protease regulatory subunit 6A	49	0	1	2	2	1	1	1.00	0.98	1.38	0.58	1.39
Prolyl endopeptidase	81	2	3	2	2	2	3	1.21	0.04	1.69	0.39	1.39
Prolow-density lipoprotein receptor-related protein 1	505	2	2	1	6	11	11	0.47	0.56	0.65	0.48	1.39
Retinoid-inducible serine carboxypeptidase	51	1	3	2	1	0	0	0.94	0.94	1.32	1.51	1.40
Bifunctional purine biosynthesis protein PURH	64	1	4	2	0	0	0	0.75	0.75	1.05	1.82	1.40
Calpain small subunit 1	28	2	4	3	2	1	2	3.47	1.65	4.85	2.03	1.40
DnaJ homolog subfamily C member 3	57	1	0	1	2	1	2	0.86	0.03	1.20	1.04	1.40
Cytoplasmic dynein 1 heavy chain 1	532	8	12	17	19	22	32	1.46	0.69	2.03	1.04	1.40
Solute carrier organic anion transporter family member 1B2	77	19	21	6	16	12	13	7.89	4.14	11.02	2.47	1.40

Lipoma-preferred partner homolog	66	1	1	1	1	1	2	0.74	0.02	1.04	0.48	1.40
Guanidinoacetate N-methyltransferase	26	1	3	5	3	3	6	5.65	3.67	7.92	3.67	1.40
Glutathione S-transferase theta-1	27	5	5	3	8	3	2	6.66	2.04	9.37	5.49	1.41
Serine/arginine repetitive matrix protein 2	295	0	0	1	0	2	4	0.17	0.17	0.24	0.41	1.41
Peroxisomal trans-2-enoyl-CoA reductase	32	8	7	9	13	6	11	11.72	1.98	16.51	4.90	1.41
Mannosyl-oligosaccharide glucosidase	92	1	0	2	3	0	1	0.52	0.52	0.74	0.83	1.41
3-hydroxyacyl-CoA dehydrogenase type-2	27	17	28	17	22	21	25	33.46	5.37	47.32	5.78	1.41
Hydroxymethylglutaryl-CoA lyase, mitochondrial	34	4	3	1	5	3	3	3.88	2.22	5.49	1.65	1.42
Phospholipid hydroperoxide glutathione peroxidase, mitochondrial	22	0	2	3	1	0	1	2.17	3.76	3.09	1.29	1.42
Eukaryotic translation initiation factor 4 gamma 1	176	7	12	12	10	9	16	2.60	0.66	3.69	0.99	1.42
Rab GDP dissociation inhibitor beta	51	6	8	3	6	7	8	5.18	2.15	7.36	1.27	1.42
Transmembrane emp24 domain-containing protein 2	23	2	3	2	3	4	5	5.75	2.70	8.19	2.83	1.42
Ubiquitin carboxyl-terminal hydrolase 47	157	1	2	2	0	0	2	0.31	0.30	0.44	0.38	1.43
Aspartoacylase-2	35	2	3	3	4	3	4	3.75	0.85	5.36	0.92	1.43
NADH dehydrogenase [ubiquinone] 1 beta subcomplex subunit 8, mitochondrial	22	1	2	1	3	3	2	3.78	2.75	5.41	1.26	1.43
Coiled-coil-helix-coiled-coil-helix domain-containing protein 3, mitochondrial	26	1	3	1	2	3	2	3.20	2.33	4.58	1.07	1.43
Carnitine O-palmitoyltransferase 1, liver isoform	88	10	14	7	16	11	9	5.23	1.31	7.51	1.92	1.44
Ras-related protein Rab-11B	24	2	2	3	4	3	5	5.47	1.24	7.85	3.42	1.44
40S ribosomal protein SA	33	5	7	5	8	3	3	6.42	1.54	9.22	3.91	1.44
T-complex protein 1 subunit alpha	60	5	5	4	8	4	5	3.55	0.45	5.10	1.39	1.44
Peroxisomal acyl-coenzyme A oxidase 2	77	7	7	2	5	4	6	2.77	1.59	3.98	0.67	1.44
Protein transport protein Sec31A	134	10	11	6	10	5	8	2.56	0.94	3.68	0.50	1.44
Sulfite oxidase, mitochondrial	61	7	10	5	8	6	7	4.84	0.85	6.96	1.15	1.44
Arylamine N-acetyltransferase 2	34	1	1	1	3	3	3	2.45	1.78	3.52	1.77	1.44
60S ribosomal protein L8	28	5	4	4	8	4	6	7.60	0.97	10.95	3.59	1.44
Cofilin-1	19	7	8	8	10	6	11	18.05	2.02	26.05	4.67	1.44

Elongation factor 2	95	21	29	17	29	23	27	10.55	1.90	15.23	0.22	1.44
Transforming protein RhoA	22	0	0	2	4	3	3	3.77	3.51	5.44	4.81	1.44
Glutamate--cysteine ligase regulatory subunit	31	0	4	3	2	2	1	2.64	2.38	3.81	2.45	1.44
Major urinary protein 6	21	1	4	6	8	8	9	11.80	8.70	17.11	6.73	1.45
Acetyl-CoA acetyltransferase, mitochondrial	45	16	25	18	18	17	28	18.56	1.10	26.95	6.40	1.45
Glutaryl-CoA dehydrogenase, mitochondrial	49	7	7	6	12	7	9	6.70	0.75	9.73	2.56	1.45
Serum paraoxonase/arylesterase 1	40	2	3	2	3	1	1	2.03	0.66	2.95	1.42	1.45
5-oxoprolinase	138	6	7	5	8	4	6	1.78	0.32	2.59	0.31	1.46
Eukaryotic translation initiation factor 4B	69	1	3	3	3	1	1	1.17	0.78	1.71	0.82	1.46
40S ribosomal protein S5	23	2	2	2	3	1	2	3.53	1.14	5.17	1.20	1.46
Molybdenum cofactor biosynthesis protein 1	70	0	1	3	3	2	3	1.17	1.05	1.71	0.86	1.46
Protein-glutamine gamma-glutamyltransferase 2	77	5	4	3	5	2	5	2.12	0.94	3.10	0.43	1.47
Cytochrome b-c1 complex subunit 1, mitochondrial	53	3	5	5	5	2	4	3.06	1.32	4.49	0.44	1.47
Glutamate--cysteine ligase catalytic subunit	73	5	9	6	6	6	9	3.82	0.44	5.61	1.29	1.47
Ras GTPase-activating-like protein IQGAP2	181	26	44	28	33	21	29	6.77	0.76	9.95	2.02	1.47
26S protease regulatory subunit 7	49	2	1	2	3	1	3	1.66	0.54	2.44	1.23	1.47
3-ketoacyl-CoA thiolase A, peroxisomal	44	15	21	15	17	18	30	17.91	2.55	26.46	8.52	1.48
Inter-alpha-trypsin inhibitor heavy chain H1	101	3	2	1	1	1	4	0.81	0.55	1.19	0.81	1.48
von Willebrand factor A domain-containing protein 5A	87	1	2	3	2	1	3	0.93	0.62	1.38	0.38	1.48
40S ribosomal protein S7	22	5	11	7	15	13	10	18.80	10.03	27.80	5.63	1.48
Complement component 1 Q subcomponent-binding protein, mitochondrial	31	0	0	4	4	1	3	2.60	3.19	3.86	3.41	1.48
Ceruloplasmin	121	2	4	4	5	1	1	0.94	0.59	1.39	0.85	1.49
Coatomer subunit zeta-1	20	3	4	2	5	4	4	7.43	2.71	11.06	1.33	1.49
Serine protease inhibitor A3N	47	0	3	0	2	4	1	1.45	2.51	2.16	1.05	1.49
4-aminobutyrate aminotransferase, mitochondrial	56	1	3	3	2	3	5	2.05	1.03	3.07	1.49	1.49
Proteasome subunit beta type-2	23	2	6	3	5	6	5	7.92	4.76	11.84	1.15	1.49
General vesicular transport factor p115	107	4	2	2	5	1	3	1.06	0.69	1.59	0.71	1.49
Cullin-associated NEDD8-dissociated protein 1	136	6	5	4	6	4	9	1.68	0.40	2.52	0.86	1.50
Valyl-tRNA synthetase	140	5	2	5	9	4	9	1.63	0.15	2.45	1.50	1.50

60S ribosomal protein L13	24	3	5	3	6	5	5	7.56	2.65	11.34	1.08	1.50
Putative pre-mRNA-splicing factor ATP-dependent RNA helicase DHX15	91	0	1	0	1	2	1	0.37	0.65	0.56	0.02	1.50
Protein-L-isoaspartate(D-aspartate) O-methyltransferase	25	0	1	0	1	2	1	1.36	2.35	2.04	0.06	1.50
3 beta-hydroxysteroid dehydrogenase type 7	41	0	1	0	1	2	1	0.83	1.44	1.25	0.03	1.50
ADP-ribosylation factor 5	21	5	8	2	11	12	9	15.09	12.66	22.70	3.57	1.50
D-beta-hydroxybutyrate dehydrogenase, mitochondrial	38	13	15	10	15	8	15	13.32	3.02	20.17	0.54	1.51
Glucokinase	52	1	2	0	1	1	0	0.64	0.55	0.97	0.97	1.52
Cytosolic non-specific dipeptidase	53	1	3	0	0	1	0	0.63	0.54	0.95	1.65	1.52
Carbonic anhydrase 2	29	5	6	5	10	5	6	8.47	0.28	12.88	3.86	1.52
Protein CREG1	24	0	0	1	3	1	0	1.37	1.19	2.09	3.63	1.53
Signal transducer and activator of transcription 1	87	0	0	0	1	2	2	0.39	0.68	0.60	0.61	1.53
Chromobox protein homolog 3	21	0	0	0	1	2	2	1.62	2.80	2.47	2.51	1.53
Mitochondrial carnitine/acylcarnitine carrier protein	33	4	6	5	6	6	10	7.47	1.70	11.42	3.94	1.53
Ras-related protein Rab-5B	24	1	2	1	3	2	1	2.76	1.29	4.22	2.04	1.53
Aldehyde dehydrogenase, cytosolic 1	55	6	7	6	10	7	11	5.67	0.71	8.69	2.10	1.53
Lon protease homolog, mitochondrial	106	6	8	9	16	8	10	3.55	0.70	5.45	1.94	1.53
Cathepsin Z	34	1	0	0	2	1	1	0.98	0.85	1.50	1.48	1.54
Annexin A5	36	9	10	7	12	5	9	9.51	2.55	14.64	1.88	1.54
V-type proton ATPase catalytic subunit A	68	2	3	1	3	1	0	0.96	0.41	1.48	1.28	1.54
Malectin	32	1	3	1	1	2	2	2.07	0.97	3.19	1.58	1.55
Probable 2-oxoglutarate dehydrogenase E1 component DHKTD1, mitochondrial	103	6	9	10	10	3	9	2.99	1.58	4.63	0.24	1.55
UPF0027 protein C22orf28 homolog	55	2	2	0	1	0	0	0.59	1.02	0.91	0.92	1.55
Multifunctional protein ADE2	47	2	2	0	1	0	0	0.69	1.20	1.07	1.07	1.55
Protein-glutamine gamma-glutamyltransferase K	90	2	3	0	0	0	0	0.36	0.62	0.56	0.97	1.55
Betaine--homocysteine S-methyltransferase 1	45	70	88	56	88	61	103	68.10	8.13	105.8	12.84	1.55
Very long-chain specific acyl-CoA dehydrogenase, mitochondrial	71	14	20	14	27	10	10	8.73	1.35	13.57	5.87	1.55
Myosin regulatory light chain 12B	20	2	5	3	3	3	4	6.56	1.49	10.22	2.54	1.56
Ras-related protein Rab-6A	24	1	0	0	1	1	2	1.38	1.20	2.16	2.20	1.56

Low molecular weight phosphotyrosine protein phosphatase	18	1	3	1	0	0	0	1.79	1.55	2.80	4.85	1.57
Protein DDI1 homolog 2	45	3	3	0	0	1	3	1.46	1.65	2.29	1.98	1.57
Cathepsin B	37	4	9	3	8	8	6	6.72	3.79	10.55	1.87	1.57
Keratin, type I cytoskeletal 18	48	6	5	2	4	2	6	3.40	2.32	5.34	1.20	1.57
Serum paraoxonase/lactonase 3	39	2	1	0	1	0	1	0.83	1.44	1.31	0.04	1.58
Endoplasmic reticulum resident protein 44	47	2	1	0	1	0	1	0.69	1.20	1.09	0.03	1.58
Src substrate cortactin	61	0	1	1	0	1	2	0.54	0.47	0.85	0.86	1.58
Isocitrate dehydrogenase [NAD] subunit alpha, mitochondrial	40	0	1	1	0	1	2	0.82	0.71	1.30	1.32	1.58
Kynurenine 3-monooxygenase	55	4	5	3	3	1	4	2.36	1.32	3.72	0.92	1.58
Calcyclin-binding protein	27	2	2	2	3	0	1	2.38	2.06	3.76	1.82	1.58
28 kDa heat- and acid-stable phosphoprotein	21	0	1	2	2	0	0	1.52	2.63	2.39	2.39	1.58
Glutaredoxin-related protein 5, mitochondrial	16	0	1	2	2	0	0	1.99	3.45	3.14	3.14	1.58
Alpha-aminoadipic semialdehyde dehydrogenase	56	9	12	6	9	12	20	7.96	2.90	12.56	5.59	1.58
Serine--pyruvate aminotransferase, mitochondrial	46	1	4	3	1	0	1	1.39	1.59	2.21	1.88	1.58
ATPase family AAA domain-containing protein 3	67	1	0	1	2	0	1	0.48	0.42	0.76	0.75	1.59
Alanine--glyoxylate aminotransferase 2, mitochondrial	57	3	1	4	5	1	6	2.27	1.26	3.61	2.43	1.59
Ras-related protein Rab-14	24	3	6	2	6	4	2	6.19	2.26	9.85	4.73	1.59
ATP-binding cassette sub-family D member 3	75	12	21	10	21	13	12	7.67	1.24	12.20	3.26	1.59
Carbonic anhydrase 3	29	38	65	55	77	46	71	78.43	13.64	125.1	10.83	1.59
Vigilin	142	28	29	19	31	10	27	6.53	3.00	10.42	0.49	1.60
Hemopexin	51	5	4	1	0	0	5	1.90	2.53	3.04	2.70	1.60
Pyruvate dehydrogenase E1 component subunit beta, mitochondrial	39	6	6	1	5	4	6	4.65	3.17	7.43	0.88	1.60
Protein tyrosine phosphatase-like protein PTPLAD1	43	0	0	2	2	0	1	0.74	1.28	1.19	1.17	1.60
Splicing factor, proline- and glutamine-rich	75	3	5	3	7	3	2	1.97	0.07	3.15	1.65	1.60
Probable ubiquitin carboxyl-terminal hydrolase FAF-X	291	2	6	3	2	6	9	0.63	0.38	1.00	0.64	1.60
Glutathione S-transferase Mu 3	26	2	5	2	4	3	2	4.43	1.26	7.16	2.86	1.61
Transcription factor A, mitochondrial	28	1	1	1	0	0	2	1.15	0.99	1.85	1.88	1.61

Long-chain-fatty-acid--CoA ligase 1	78	17	35	20	27	22	30	12.41	1.89	20.09	2.60	1.62
Heterogeneous nuclear ribonucleoprotein F	46	1	3	1	4	3	1	1.81	1.31	2.93	1.64	1.62
Carboxylesterase 3	62	18	22	12	23	11	19	10.81	2.87	17.55	1.28	1.62
Heterogeneous nuclear ribonucleoprotein U	88	3	4	5	8	1	2	1.65	1.07	2.68	1.72	1.63
Cytochrome P450 2C54	56	2	2	0	4	3	2	1.49	1.38	2.42	1.01	1.63
Calpastatin	85	2	3	2	4	3	4	1.36	0.38	2.21	0.38	1.63
Large proline-rich protein BAT3	121	0	0	2	1	0	2	0.26	0.46	0.43	0.44	1.63
NADH dehydrogenase [ubiquinone] 1 alpha subcomplex subunit 9, mitochondrial	43	3	3	2	5	2	3	2.66	0.64	4.34	1.30	1.63
Inosine triphosphate pyrophosphatase	22	1	3	1	3	3	2	3.78	2.75	6.17	1.19	1.63
UDP-glucose 6-dehydrogenase	55	3	6	5	10	4	3	3.57	0.86	5.83	3.15	1.63
Cordon-bleu protein-like 1	137	11	14	5	7	5	12	2.50	1.22	4.11	1.36	1.64
Sideroflexin-1	36	2	3	2	2	3	6	3.20	0.91	5.26	3.13	1.64
Proteasome subunit beta type-1	26	5	6	3	7	4	6	7.57	1.93	12.44	0.98	1.64
Dihydrodipicolinate synthase-like, mitochondrial	35	5	7	6	12	4	5	6.99	1.19	11.61	5.03	1.66
Fermitin family homolog 2	78	0	3	0	1	3	1	0.65	1.13	1.09	0.74	1.66
Keratin, type II cytoskeletal 2 epidermal	71	5	5	3	8	2	3	2.29	1.02	3.81	1.73	1.66
Dimethylaniline monooxygenase [N-oxide-forming] 1	60	1	3	2	2	2	3	1.37	0.49	2.28	0.52	1.66
Radixin	69	8	9	4	7	3	8	3.54	1.84	5.93	0.75	1.67
NADPH--cytochrome P450 reductase	77	7	13	11	14	10	18	5.96	1.33	9.99	2.04	1.68
Glycerol-3-phosphate dehydrogenase, mitochondrial	81	4	5	5	5	1	6	1.99	1.21	3.37	0.46	1.69
Uncharacterized protein C1orf50 homolog	22	1	2	3	2	1	4	3.68	2.46	6.24	2.89	1.69
Probable phospholipid-transporting ATPase 11C	129	8	17	8	13	9	11	3.18	0.33	5.40	1.11	1.70
Complement C4-B	193	9	15	3	10	8	8	1.71	0.84	2.90	0.90	1.70
S-phase kinase-associated protein 1	19	0	2	2	5	4	3	5.26	5.37	8.95	4.00	1.70
Cathepsin H	37	1	3	1	2	1	0	1.33	0.04	2.27	2.08	1.71
Moesin	68	0	0	1	3	2	2	0.73	0.75	1.26	1.14	1.71
Heterogeneous nuclear ribonucleoprotein A1	34	0	1	1	2	2	2	1.47	1.50	2.51	0.90	1.71
NADH dehydrogenase [ubiquinone] iron-sulfur protein 4, mitochondrial	20	2	3	1	4	3	3	4.97	2.63	8.50	1.35	1.71
Transcription factor BTF3	22	0	7	5	7	7	6	9.03	8.27	15.46	0.94	1.71
Signal recognition particle receptor subunit beta	30	0	2	2	3	1	0	1.63	1.59	2.79	2.56	1.72

AP-2 complex subunit alpha-1	108	1	1	0	1	2	3	0.46	0.47	0.80	0.58	1.72
LETM1 and EF-hand domain-containing protein 1, mitochondrial	83	3	4	4	3	1	6	1.56	0.86	2.68	1.02	1.72
Probable N-acetyltransferase CML1	25	1	2	1	2	1	1	1.97	0.07	3.39	1.11	1.72
Coronin-1B	54	1	2	1	2	1	1	0.91	0.03	1.57	0.51	1.72
Cytochrome P450 2E1	57	9	12	9	15	11	21	8.36	1.29	14.41	4.52	1.72
60S ribosomal protein L11	20	3	10	4	10	12	12	15.82	12.85	27.31	3.73	1.73
Tubulin alpha-4A chain	50	1	2	1	1	1	2	0.98	0.03	1.71	0.61	1.74
L-xylulose reductase	26	3	2	4	9	2	4	5.63	1.72	9.79	6.92	1.74
Dihydropyrimidinase	57	6	9	4	12	6	6	4.61	1.10	8.03	2.52	1.74
Sulfotransferase family cytosolic 1B member 1	35	0	1	2	2	1	2	1.40	1.37	2.44	0.87	1.75
Retinal dehydrogenase 1	54	46	64	30	59	25	49	30.54	9.56	54.13	5.97	1.77
6-phosphofructokinase, liver type	85	1	1	2	2	0	2	0.57	0.56	1.00	0.36	1.78
Serine/threonine-protein kinase MRCK beta	195	0	3	1	2	3	2	0.34	0.40	0.61	0.14	1.78
Phosphopantothenate--cysteine ligase	34	0	0	2	1	1	4	1.44	1.41	2.56	3.24	1.78
Mitochondrial import inner membrane translocase subunit Tim23	22	1	2	0	2	3	3	3.05	3.55	5.44	1.51	1.78
ADP-ribosylation factor-like protein 8B	22	1	2	0	2	3	3	3.05	3.55	5.44	1.51	1.78
Coatomer subunit alpha	138	9	14	5	12	8	12	2.62	0.78	4.69	0.38	1.79
Lamin-A/C	74	1	5	3	6	4	3	1.78	1.06	3.20	0.98	1.80
Cytochrome P450 2C50	56	4	5	1	6	3	3	2.35	1.35	4.23	1.30	1.80
UDP-glucuronosyl-transferase 2A3	61	2	5	3	6	3	3	2.15	0.49	3.89	1.19	1.81
Uncharacterized protein KIAA0564 homolog	213	13	24	13	20	16	29	3.24	0.51	5.86	1.24	1.81
Glucosamine 6-phosphate N-acetyltransferase	21	2	5	2	7	5	4	7.11	4.36	12.93	3.45	1.82
Apolipoprotein A-I	31	9	12	2	8	6	10	9.03	5.57	16.49	3.29	1.83
Ornithine aminotransferase, mitochondrial	48	9	16	8	16	9	14	8.88	0.82	16.30	0.81	1.84
Complement factor H	139	2	3	2	3	0	1	0.46	0.40	0.85	0.41	1.84
Phosphatidylcholine transfer protein	25	2	2	1	1	1	4	2.61	1.11	4.82	3.28	1.84
NADH dehydrogenase [ubiquinone] 1 alpha subcomplex subunit 10, mitochondrial	41	0	2	3	2	1	3	1.58	1.77	2.92	0.81	1.85
Probable D-lactate dehydrogenase, mitochondrial	52	0	3	2	3	3	3	1.59	1.49	2.95	0.08	1.85
GTPase-activating protein and VPS9 domain-containing protein 1	162	1	6	3	1	1	2	0.50	0.33	0.94	0.82	1.88
3-mercaptopyruvate sulfurtransferase	33	6	9	4	6	2	7	5.91	2.88	11.35	2.30	1.92

Proteasome-associated protein ECM29 homolog	204	1	5	4	3	2	5	0.56	0.35	1.09	0.30	1.95
Protein BAT2-like 2	309	0	0	0	2	1	0	0.06	0.10	0.11	0.19	1.97
Sigma 1-type opioid receptor	25	0	0	0	2	1	0	0.68	1.18	1.34	2.32	1.97
Extracellular matrix protein FRAS1	442	0	2	0	0	1	0	0.04	0.07	0.08	0.13	1.98
Leucyl-cystinyl aminopeptidase	117	0	2	0	0	1	0	0.15	0.25	0.29	0.50	1.98
Major urinary protein 2	21	17	46	32	70	46	67	74.64	36.19	148.6	33.21	1.99
Serine/arginine repetitive matrix protein 1	107	1	2	0	2	1	0	0.31	0.27	0.63	0.54	2.02
Ubiquitin-conjugating enzyme E2 N	17	1	2	0	2	1	0	1.95	1.69	3.95	3.42	2.02
Transaldolase	37	0	3	1	2	2	1	1.35	1.38	2.74	1.33	2.03
Acyl-coenzyme A synthetase ACSM3, mitochondrial	66	0	3	1	1	1	0	0.50	0.43	1.02	1.17	2.04
Asialoglycoprotein receptor1	33	1	2	0	1	1	1	1.01	0.87	2.06	0.86	2.04
3-oxoacyl-[acyl-carrier-protein] synthase, mitochondrial	49	1	2	0	1	1	1	0.68	0.59	1.39	0.58	2.04
Perilipin-2	47	0	2	1	2	2	2	1.06	1.09	2.17	0.06	2.05
Peroxisomal Lon protease homolog 2	95	0	2	1	2	2	2	0.53	0.54	1.08	0.03	2.05
NADH dehydrogenase [ubiquinone] 1 beta subcomplex subunit 9	22	1	2	1	4	3	4	3.78	2.75	7.77	2.77	2.05
60S ribosomal protein L12	18	0	1	2	5	4	6	5.55	5.67	11.44	7.70	2.06
7-alpha-hydroxycholest-4-en-3-one 12-alpha-hydroxylase	58	0	0	0	0	1	2	0.29	0.51	0.61	1.05	2.07
Molybdopterin synthase catalytic subunit	21	0	0	0	0	1	2	0.81	1.40	1.67	2.90	2.07
Clathrin light chain B	25	1	2	0	0	0	0	0.65	1.12	1.34	2.33	2.07
Proteasome activator complex subunit 4	211	1	2	0	0	0	0	0.08	0.13	0.16	0.28	2.07
Neutrophil gelatinase-associated lipocalin	23	1	2	0	0	0	0	0.71	1.22	1.46	2.53	2.07
Ras-related protein Rap-2c	21	1	0	1	4	1	2	2.34	0.08	4.86	4.79	2.08
Enoyl-CoA hydratase domain-containing protein1	35	0	0	1	2	2	4	1.43	1.46	2.96	3.01	2.08
Band 3 anion transport protein	103	1	3	1	1	1	2	0.48	0.02	0.99	0.49	2.08
Myosin-Ib	129	1	1	2	5	2	4	0.64	0.23	1.32	0.82	2.08
Phospholipase A-2-activating protein	87	0	1	1	1	1	2	0.38	0.33	0.79	0.37	2.08
NADH dehydrogenase [ubiquinone] iron-sulfur protein 7, mitochondrial	25	1	1	1	3	0	0	1.29	1.11	2.68	3.07	2.08
Kynureninase	52	0	2	1	0	1	2	0.63	0.55	1.32	1.14	2.09
Aminoacyl tRNA synthetase complex-interacting multifunctional protein 1	34	0	2	1	0	1	2	0.97	0.84	2.02	1.75	2.09

Ankyrin-1	204	1	2	1	2	0	0	0.16	0.14	0.33	0.28	2.09
Carboxymethylenebutenolidase homolog	28	4	5	3	6	5	13	7.06	1.99	14.74	8.47	2.09
FGGY carbohydrate kinase domain-containing protein	60	0	0	1	2	0	0	0.27	0.46	0.56	0.97	2.10
Destrin	19	1	2	2	3	0	1	2.53	2.52	5.34	2.58	2.11
Ras-related protein Rap-1b	21	1	2	3	3	2	7	4.67	2.26	9.85	6.79	2.11
Alpha-1-antitrypsin 1-4	46	1	1	1	2	0	1	0.70	0.61	1.48	0.62	2.11
Stomatin-like protein 2	38	1	2	1	1	0	1	0.85	0.73	1.79	0.75	2.11
Keratin, type II cytoskeletal 8	55	5	3	2	4	2	11	2.67	1.52	5.65	4.26	2.11
Calcium-regulated heat stable protein 1	16	0	1	2	2	0	1	1.99	3.45	4.24	1.77	2.13
Ras GTPase-activating protein-binding protein 1	52	1	1	1	1	0	2	0.62	0.54	1.32	0.61	2.14
Acyl-coenzyme A synthetase ACSM5, mitochondrial	64	1	2	1	0	0	2	0.50	0.44	1.07	0.93	2.14
Coproporphyrinogen-III oxidase, mitochondrial	50	1	4	3	0	0	4	1.28	1.46	2.75	2.38	2.15
Heterogeneous nuclear ribonucleoprotein D0	38	0	0	2	2	0	2	0.84	1.45	1.81	1.57	2.15
Cold shock domain-containing protein E1	89	0	1	2	1	0	2	0.36	0.62	0.77	0.36	2.15
Neurolysin, mitochondrial	80	1	0	0	0	0	2	0.20	0.35	0.44	0.76	2.17
Cytochrome P450 2A12	56	1	5	3	7	3	3	2.05	1.03	4.53	1.73	2.21
DNA damage-binding protein 1	127	3	5	2	7	2	3	0.90	0.22	2.00	0.76	2.22
Glycine N-acyltransferase-like protein	34	8	9	5	10	3	15	7.66	3.52	17.12	5.36	2.24
Vacuolar protein-sorting-associated protein 25	21	0	2	1	5	4	4	4.00	5.08	8.93	3.71	2.24
GDH/6PGL endoplasmic bifunctional protein	89	4	6	1	2	2	7	1.29	0.83	2.89	1.57	2.24
Ferritin heavy chain	21	2	5	2	5	2	3	4.68	0.16	10.49	2.57	2.24
E3 ubiquitin-protein ligase UBR4	572	0	0	0	0	5	11	0.15	0.26	0.34	0.59	2.27
Dihydrofolate reductase	22	2	2	1	5	2	4	3.74	1.36	8.53	3.54	2.28
Ubiquitin carboxyl-terminal hydrolase 5	96	3	4	4	7	2	9	1.52	0.47	3.57	1.42	2.34
Chloride intracellular channel protein 1	27	0	2	1	4	2	1	1.85	1.89	4.38	2.80	2.37
Keratin, type I cytoskeletal 10	58	2	3	2	3	0	3	1.11	0.96	2.64	0.07	2.38
AP-1 complex subunit gamma-1	91	0	3	2	4	1	0	0.54	0.53	1.29	1.15	2.40
Multidrug resistance-associated protein 6	165	1	5	2	3	3	6	0.60	0.32	1.45	0.51	2.42
Spectrin beta chain, erythrocyte	245	1	4	1	1	1	2	0.20	0.01	0.49	0.31	2.42
Regulator of microtubule dynamics protein 3	52	0	3	2	2	1	2	0.94	0.92	2.29	0.54	2.43
Ubiquitin carboxyl-terminal hydrolase 14	56	0	0	1	4	1	1	0.59	0.51	1.51	1.86	2.57

3-ketoacyl-CoA thiolase B, peroxisomal	44	2	5	2	6	4	9	3.01	1.41	7.78	2.65	2.59
Eukaryotic translation initiation factor 3 subunit C	106	1	7	2	2	1	1	0.61	0.25	1.59	1.52	2.59
1,2-dihydroxy-3-keto-5-methylthiopentene dioxygenase	22	1	1	0	1	1	3	1.51	1.31	3.92	2.83	2.60
60S ribosomal protein L10	25	0	4	3	4	1	2	2.59	2.91	6.77	2.21	2.61
Vesicle-fusing ATPase	83	1	2	1	2	0	1	0.39	0.34	1.02	0.33	2.63
Leucyl-tRNA synthetase, cytoplasmic	134	2	3	1	1	1	6	0.49	0.21	1.29	1.00	2.64
Catenin alpha-1	100	2	3	2	4	0	3	0.64	0.56	1.70	0.27	2.64
CD166 antigen	65	1	2	1	1	0	2	0.49	0.43	1.31	0.47	2.66
U2 small nuclear ribonucleoprotein A'	28	2	1	0	1	0	3	1.16	2.01	3.08	2.22	2.66
Isoleucyl-tRNA synthetase, mitochondrial	113	1	4	1	4	3	5	0.74	0.54	1.96	0.32	2.67
Calcineurin-like phosphoesterase domain-containing protein 1	35	0	1	2	2	0	2	0.91	1.58	2.44	0.87	2.68
E3 SUMO-protein ligase RanBP2	341	0	2	2	1	0	2	0.09	0.16	0.25	0.09	2.68
Ectonucleoside triphosph-ate diphosphohydrolase 5	47	2	1	0	0	0	4	0.69	1.20	1.85	2.34	2.68
Alpha-actinin-1	103	2	3	1	5	2	5	0.80	0.29	2.15	0.60	2.69
Cystathionine beta-synthase	62	4	4	2	8	1	6	1.83	1.19	4.94	1.62	2.70
Apoptosis regulator BAX	21	0	2	1	2	2	4	2.38	2.43	6.54	3.03	2.75
Annexin A2	39	2	1	0	3	1	4	1.27	1.25	3.52	2.08	2.78
Alanine aminotransferase 1	55	2	4	0	0	1	4	0.90	0.88	2.50	2.17	2.78
28S ribosomal protein S23, mitochondrial	20	0	1	0	2	1	0	0.85	1.47	2.51	2.51	2.96
Septin-9	66	0	2	0	1	1	0	0.26	0.45	0.76	0.76	2.96
Retinol-binding protein 4	23	0	3	0	0	1	0	0.74	1.28	2.19	3.79	2.96
Liver carboxylesterase 1	63	3	12	2	3	2	5	1.82	0.43	5.39	3.75	2.96
Carbonic anhydrase 1	28	0	3	0	2	2	1	1.21	2.10	3.62	1.76	2.98
COMM domain-containing protein 3	22	0	2	0	0	1	1	0.77	1.34	2.32	2.29	3.01
Aminopeptidase N	110	4	9	3	6	1	8	1.18	0.66	3.56	0.73	3.02
Serrate RNA effector molecule homolog	100	0	0	0	1	1	2	0.17	0.29	0.52	0.53	3.05
39S ribosomal protein L16, mitochondrial	29	0	1	0	0	1	2	0.59	1.02	1.79	1.82	3.05
39S ribosomal protein L11, mitochondrial	21	0	1	0	0	1	2	0.81	1.40	2.47	2.51	3.05
GTP cyclohydrolase 1	27	0	1	0	0	1	2	0.63	1.09	1.92	1.95	3.05
Selenoprotein S	22	0	1	0	0	1	2	0.77	1.34	2.36	2.40	3.05
Derlin-2	28	1	2	0	2	1	2	1.19	1.03	3.65	0.10	3.08
Cytochrome P450 2A4	57	1	0	0	3	0	0	0.28	0.49	0.88	1.53	3.10
Carcinoembryonic antigen-related cell adhesion molecule 1	57	1	2	0	1	0	0	0.28	0.49	0.88	0.88	3.10
Nicotinamide phosphoribosyltransferase	55	1	3	0	0	0	0	0.29	0.51	0.92	1.59	3.11

Keratin, type II cytoskeletal 1	66	1	4	2	6	3	8	1.50	0.79	4.67	1.67	3.11
Transmembrane and coiled-coil domain-containing protein 1	21	0	1	1	2	1	3	1.57	1.36	4.90	2.57	3.13
Gamma-aminobutyric acid receptor-associated protein	14	1	2	1	3	0	1	2.30	1.99	7.24	3.50	3.15
Dolichyl-diphosphooligosaccharide--protein glycosyltransferase subunit STT3A	81	1	2	1	3	0	1	0.40	0.34	1.25	0.61	3.15
Epidermal growth factor receptor kinase substrate 8-like protein 2	82	1	2	0	0	0	1	0.20	0.34	0.62	0.61	3.15
Eukaryotic translation initiation factor 3 subunit F	38	1	2	0	0	0	1	0.43	0.74	1.35	1.33	3.15
Seryl-tRNA synthetase, cytoplasmic	58	0	1	1	2	0	0	0.27	0.48	0.87	0.87	3.16
NADH dehydrogenase [ubiquinone] 1 beta subcomplex subunit 11, mitochondrial	17	0	2	1	1	0	0	0.94	1.62	2.96	2.96	3.16
Golgi SNAP receptor complex member 2	25	0	2	1	1	0	0	0.64	1.10	2.01	2.01	3.16
28S ribosomal protein S15, mitochondrial	29	1	1	0	0	0	2	0.56	0.97	1.79	1.82	3.20
26S proteasome non-ATPase regulatory subunit 9	25	0	0	1	2	0	1	0.64	1.10	2.04	2.01	3.20
Phosphatidate phosphatase LPIN1	102	0	0	1	1	0	2	0.16	0.27	0.51	0.52	3.26
Actin-related protein 2/3 complex subunit 4	20	0	4	1	1	2	5	2.50	2.55	8.59	5.48	3.44
Regulator of nonsense transcripts 1	124	2	5	1	5	0	0	0.39	0.39	1.35	1.17	3.47
Peroxisomal carnitine O-octanoyltransferase	70	1	5	2	4	0	1	0.69	0.68	2.41	1.48	3.50
Keratin, type I cytoskeletal 14	53	1	1	0	3	1	3	0.63	0.54	2.26	1.14	3.61
Fumarylacetoacetate hydrolase domain-containing protein 2A	35	0	5	1	0	1	2	0.94	0.82	3.40	3.61	3.61
Epoxide hydrolase 1	53	1	6	2	4	1	4	1.23	0.50	4.49	1.05	3.66
Insulin-degrading enzyme	118	2	1	0	3	0	3	0.27	0.48	1.01	0.51	3.69
Disks large homolog 1	100	0	3	2	3	0	1	0.32	0.55	1.18	0.57	3.71
Fatty aldehyde dehydrogenase	54	2	6	2	6	2	10	1.82	0.06	6.98	2.41	3.83
Monoglyceride lipase	33	0	1	0	2	1	1	0.52	0.89	2.06	0.86	3.99
Transcription intermediary factor 1-beta	89	0	3	0	0	1	1	0.19	0.33	0.76	0.86	4.00
GTPase HRas	21	0	1	0	1	1	2	0.81	1.40	3.27	1.51	4.04
28S ribosomal protein S25, mitochondrial	20	0	1	0	1	1	2	0.85	1.47	3.43	1.59	4.04
Mitochondrial 2-oxodicarboxylate carrier	33	0	1	0	0	1	3	0.52	0.89	2.11	2.45	4.09

Kynurenine--oxoglutarate transaminase 3	51	1	4	0	1	1	3	0.65	0.56	2.68	1.53	4.11
Arginyl-tRNA synthetase, cytoplasmic	76	1	1	0	3	0	0	0.21	0.37	0.88	1.01	4.13
Acyl-CoA dehydrogenase family member 9, mitochondrial	69	1	0	0	3	0	1	0.24	0.41	0.98	1.11	4.18
Eukaryotic translation initiation factor 3 subunit K	25	1	1	0	2	0	1	0.65	1.12	2.71	1.13	4.18
Glutathione S-transferase A1	26	2	3	1	6	0	3	1.86	1.87	7.83	3.27	4.21
26S protease regulatory subunit 8	46	1	0	0	2	0	2	0.35	0.61	1.49	1.29	4.23
Peroxisomal membrane protein 4	24	0	2	1	1	0	1	0.66	1.15	2.83	1.19	4.26
Stromal cell-derived factor 2-like protein 1	24	0	2	1	1	0	1	0.66	1.15	2.83	1.19	4.26
28S ribosomal protein S2, mitochondrial	32	0	1	1	1	0	2	0.50	0.86	2.15	0.99	4.31
Solute carrier family 25 member 42	35	0	0	1	1	0	3	0.46	0.79	1.98	2.31	4.36
Multidrug resistance protein 2	140	1	6	2	3	1	8	0.46	0.19	2.08	0.97	4.48
Presequence protease, mitochondrial	117	0	2	0	2	1	1	0.15	0.25	0.72	0.24	4.98
Large proline-rich protein BAT2	229	0	4	0	0	1	1	0.07	0.13	0.37	0.46	4.98
Ubiquilin-1	62	0	2	0	1	1	2	0.27	0.47	1.38	0.49	5.03
Probable 10-formyltetrahydrofolate dehydrogenase ALDH1L2	102	1	1	0	3	0	1	0.16	0.28	0.83	0.56	5.22
Cutaneous T-cell lymphoma-associated antigen 5 homolog	88	1	2	0	2	0	1	0.18	0.32	0.96	0.31	5.22
Antithrombin-III	52	0	4	1	1	0	0	0.31	0.53	1.61	2.02	5.26
26S proteasome non-ATPase regulatory subunit 14	35	0	1	1	3	0	1	0.46	0.79	2.42	1.64	5.31
Aminoglycoside phosphor-transferase domain-containing protein 1	42	0	2	1	2	0	1	0.38	0.66	2.02	0.66	5.31
Uncharacterized protein KIAA0664	148	2	14	5	10	0	12	0.76	0.81	4.14	0.69	5.47
Talin-2	254	1	6	0	4	1	1	0.13	0.11	0.73	0.49	5.58
Annexin A4	36	2	5	0	2	0	4	0.90	1.56	5.21	2.17	5.79
Partner of Y14 and mago	23	1	0	0	5	0	1	0.71	1.22	4.41	5.76	6.25
Caprin-1	78	1	1	0	3	0	2	0.21	0.36	1.31	0.64	6.30
Puromycin-sensitive aminopeptidase	103	1	1	0	3	0	2	0.16	0.27	0.99	0.49	6.30
Palmitoyl-protein thioesterase 1	34	1	2	0	1	0	3	0.48	0.83	3.03	1.59	6.35
Thyroid hormone receptor-associated protein 3	108	0	2	1	3	0	2	0.15	0.26	1.10	0.26	7.46

N(G),N(G)-dimethylarginine dimethylaminohydrolase 1	31	0	3	0	3	1	3	0.55	0.95	4.95	0.13	9.02
Protein transport protein Sec24A	119	0	5	1	5	0	3	0.13	0.23	1.85	0.45	13.83
Canalicular multispecific organic anion transporter 2	169	0	7	0	2	0	5	0.00	0.00	1.41	0.76	∞
Endoplasmic reticulum aminopeptidase 1	107	0	3	0	2	0	2	0.00	0.00	1.11	0.26	∞
Tuberin	202	0	1	0	1	0	2	0.00	0.00	0.34	0.16	∞
Isoleucyl-tRNA synthetase, cytoplasmic	144	0	2	0	3	0	4	0.00	0.00	1.07	0.38	∞
Fanconi anemia group I protein homolog	149	0	0	0	2	0	1	0.00	0.00	0.34	0.34	∞
Nascent polypeptide-associated complex subunit alpha, muscle-specific form	221	0	0	0	2	0	1	0.00	0.00	0.23	0.23	∞
Spectrin alpha chain, erythrocyte	280	0	4	0	2	0	1	0.00	0.00	0.42	0.27	∞
PERQ amino acid-rich with GYF domain-containing protein 2	149	0	2	0	0	0	1	0.00	0.00	0.34	0.34	∞
Serine/threonine-protein kinase mTOR	289	0	2	0	0	0	2	0.00	0.00	0.24	0.21	∞
Eukaryotic translation initiation factor 2 subunit 1	36	0	2	0	1	0	1	0.00	0.00	1.89	0.79	∞
Nucleoside diphosphate kinase B	17	0	3	0	2	0	1	0.00	0.00	5.97	2.89	∞
Metaxin-2	30	0	2	0	1	0	1	0.00	0.00	2.26	0.95	∞
26S proteasome non-ATPase regulatory subunit 12	53	0	2	0	0	0	2	0.00	0.00	1.30	1.12	∞
Tetratricopeptide repeat protein 35	35	0	0	0	3	0	1	0.00	0.00	1.94	2.19	∞
AP-3 complex subunit delta-1	135	0	1	0	3	0	0	0.00	0.00	0.50	0.57	∞
ATP-dependent RNA helicase A	149	0	3	0	0	0	1	0.00	0.00	0.46	0.51	∞
Hydroxyacid-oxoacid transhydrogenase, mitochondrial	50	0	1	0	1	0	2	0.00	0.00	1.37	0.64	∞
Nucleobindin-1	53	0	1	0	0	0	2	0.00	0.00	0.98	0.99	∞
CDP-diacylglycerol--inositol 3-phosphatidyltransferase	24	0	1	0	1	0	3	0.00	0.00	3.59	2.59	∞
COMM domain-containing protein 1	21	0	2	0	3	0	0	0.00	0.00	3.99	3.66	∞
39S ribosomal protein L13, mitochondrial	21	0	1	0	1	0	2	0.00	0.00	3.27	1.51	∞
Monoacylglycerol lipase ABHD6	38	0	2	0	0	0	2	0.00	0.00	1.81	1.57	∞
Lysosomal protective protein	54	0	2	0	0	0	2	0.00	0.00	1.27	1.10	∞
Mitochondrial-processing peptidase subunit alpha	58	0	1	0	2	0	0	0.00	0.00	0.87	0.87	∞

Gephyrin	83	0	1	0	0	0	3	0.00	0.00	0.84	0.97	∞
Uncharacterized protein KIAA0090	112	0	1	0	0	0	3	0.00	0.00	0.62	0.72	∞
ATP-binding cassette sub-family A member 1	254	0	2	0	0	0	1	0.00	0.00	0.20	0.20	∞
ATP-binding cassette sub-family D member 1	82	0	0	0	2	0	1	0.00	0.00	0.62	0.61	∞
Beta-actin-like protein 2	42	0	2	0	1	0	0	0.00	0.00	1.20	1.20	∞
NADPH:adrenodoxin oxidoreductase, mitochondrial	54	0	1	0	0	0	2	0.00	0.00	0.96	0.98	∞
Apoptosis inhibitor 5	57	0	1	0	2	0	0	0.00	0.00	0.88	0.88	∞
COMM domain-containing protein 9	22	0	2	0	0	0	1	0.00	0.00	2.32	2.29	∞
Eukaryotic translation initiation factor 3 subunit J	29	0	1	0	2	0	0	0.00	0.00	1.73	1.73	∞
Alpha-mannosidase 2C1	116	0	2	0	1	0	0	0.00	0.00	0.43	0.43	∞
Mitochondrial carrier homolog 1	42	0	0	0	2	0	1	0.00	0.00	1.22	1.20	∞
Acyl-CoA synthetase short-chain family member 3, mitochondrial	75	0	2	0	0	0	1	0.00	0.00	0.68	0.67	∞
HBS1-like protein	75	0	2	0	0	0	0	0.00	0.00	0.45	0.78	∞
Uncharacterized protein C1orf93 homolog	22	0	0	0	2	0	0	0.00	0.00	1.52	2.64	∞
Apoptotic chromatin condensation inducer in the nucleus	151	0	3	0	0	0	0	0.00	0.00	0.33	0.58	∞
Calpain-1 catalytic subunit	82	0	3	0	0	0	0	0.00	0.00	0.61	1.06	∞
Serine/threonine-protein kinase TAO3	105	0	0	0	3	0	0	0.00	0.00	0.48	0.83	∞
Host cell factor 1	211	0	0	0	0	0	2	0.00	0.00	0.17	0.29	∞
Acyl-coenzyme A amino acid N-acyltransferase 1	46	0	2	0	0	0	0	0.00	0.00	0.73	1.26	∞
BCL2/adenovirus E1B 19 kDa protein-interacting protein 3	21	0	0	0	0	0	2	0.00	0.00	1.67	2.90	∞
Carbonic anhydrase 5A, mitochondrial	34	0	0	0	2	0	0	0.00	0.00	0.99	1.71	∞
Coronin-7	101	0	2	0	0	0	0	0.00	0.00	0.33	0.58	∞
Cleavage and polyadenylation specificity factor subunit 1	161	0	2	0	0	0	0	0.00	0.00	0.21	0.36	∞
Probable ATP-dependent RNA helicase DDX17	72	0	0	0	2	0	0	0.00	0.00	0.47	0.81	∞
Guanine deaminase	51	0	0	0	0	0	2	0.00	0.00	0.69	1.19	∞
Hippocalcin-like protein 1	22	0	0	0	2	0	0	0.00	0.00	1.52	2.64	∞
Nck-associated protein 1	129	0	0	0	2	0	0	0.00	0.00	0.26	0.45	∞
Peroxisomal membrane protein 11B	29	0	2	0	0	0	0	0.00	0.00	1.16	2.00	∞
Splicing factor 3A subunit1	89	0	2	0	0	0	0	0.00	0.00	0.38	0.65	∞
		M #1	M #2	M #3	M #4	M #5	M #6					
∑ SAF		205.6	198.6	209.1	199.0	196.1	189.8					
